# An assessment of different electronic structure approaches for modeling time-resolved x-ray absorption spectroscopy

**DOI:** 10.1063/4.0000070

**Published:** 2021-03-12

**Authors:** Shota Tsuru, Marta L. Vidal, Mátyás Pápai, Anna I. Krylov, Klaus B. Møller, Sonia Coriani

**Affiliations:** 1 DTU Chemistry, Technical University of Denmark, Kemitorvet Building 207, DK-2800 Kgs. Lyngby, Denmark; 2 Department of Chemistry, University of Southern California, Los Angeles, California 90089, USA

## Abstract

We assess the performance of different protocols for simulating excited-state x-ray absorption spectra. We consider three different protocols based on equation-of-motion coupled-cluster singles and doubles, two of them combined with the maximum overlap method. The three protocols differ in the choice of a reference configuration used to compute target states. Maximum-overlap-method time-dependent density functional theory is also considered. The performance of the different approaches is illustrated using uracil, thymine, and acetylacetone as benchmark systems. The results provide guidance for selecting an electronic structure method for modeling time-resolved x-ray absorption spectroscopy.

## I. INTRODUCTION

Since the pioneering study by Zewail's group in the mid-1980s,^1^ ultrafast dynamics has been an active area of experimental research. Advances in light sources provide new means for probing dynamics by utilizing core-level transitions. X-ray free electron lasers (XFELs) and instruments based on high-harmonic generation (HHG) enable spectroscopic measurements on the femtosecond^2–4^ and attosecond^5–8^ time scales. Methods for investigating femtosecond dynamics can be classified into two categories: (*i*) methods that track the electronic structure as parametrically dependent on the nuclear dynamics, such as time-resolved photoelectron spectroscopy (TR-PES)^9–12^ and (*ii*) methods that directly visualize nuclear dynamics, such as ultrafast x-ray scattering^13–16^ and ultrafast electron diffraction.^12,17^ Time-resolved x-ray absorption spectroscopy (TR-XAS) belongs to the former category. Similar to x-ray photoelectron spectroscopy (XPS), XAS is also element and chemical-state specific^18^ but is able to resolve the underlying electronic states better than TR-XPS. On the other hand, TR-XPS affords photoelectron detection from all the involved electronic states with higher yield. XAS has been used to probe the local structure of bulk-solvated systems, such as in most chemical reaction systems in the lab and in cytoplasm. TR-XAS has been employed to track photo-induced dynamics in organic molecules^19–22^ and transition metal complexes.^3,23–25^ With the aid of simulations,^26^ nuclear dynamics can be extracted from experimental TR-XAS spectra.

Similar to other time-resolved experimental methods from category (*i*), interpretation of TR-XAS relies on computational methods for simulating electronic structure and nuclear wave-packet dynamics. In this context, electronic structure calculations should be able to provide the following: (1) XAS of the ground states; (2) a description of the valence-excited states involved in the dynamics; and (3) XAS of the valence-excited states.

Quantum chemistry has made major progress in simulations of XAS spectra of ground states.^27,28^ Among currently available methods, the transition-potential density functional theory (TP-DFT) with the half core-hole approximation^29,30^ is widely used to interpret the XAS spectra of ground states.^31,32^ Ehlert *et al.* extended the TP-DFT method to core excitations from valence-excited states^33^ and implemented it in PSIXAS,^34^ a plugin to the Psi4 code. TP-DFT is capable of simulating (TR-)XAS spectra of large molecules with reasonable accuracy, as long as the core-excited states can be described by a single electronic configuration. Other extensions of Kohn–Sham DFT, suitable for calculating the XAS spectra of molecules in their ground states, also exist.^35^ Linear-response (LR) time-dependent (TD) DFT, a widely used method for excited states,^36–39^ has been extended to the calculation of core-excited states^40,41^ by means of the core-valence separation (CVS) scheme,^42^ a variant of truncated single excitation space (TRNSS) approach.^43^ In the CVS scheme, configurations that do not involve core orbitals are excluded from the excitation space; this is justified because the respective matrix elements are small, owing to the localized nature of the core orbitals and the large energetic gap between the core and the valence orbitals.

Core-excitation energies calculated using TDDFT show errors up to ≈20 eV when standard exchange-correlation (xc) functionals such as B3LYP^44^ are used. The errors can be reduced by using specially designed xc-functionals, such as those reviewed in Sec. 3.4.4. of Ref. 27. Hait and Head-Gordon recently developed a square gradient minimum (SGM) algorithm for excited-state orbital optimization to obtain spin-pure restricted open-shell Kohn–Sham (ROKS) energies of core-excited states; they reported sub-eV errors in XAS transition energies.^45^

The maximum overlap method (MOM)^46^ provides access to excited-state self-consistent field (SCF) solutions and, therefore, can be used to directly compute core-level states. More importantly, MOM can be also combined with TDDFT to compute core excitations from a valence-excited state.^20,22,47^ MOM-TDDFT is an attractive method for simulating TR-XAS spectra because it is computationally cheap and may provide excitation energies consistent with the TDDFT potential energy surfaces, which are often used in the nuclear dynamics simulations. However, in MOM calculations the initial valence-excited states are independently optimized and thus not orthogonal to each other. This non-orthogonality may lead to changes in the energetic order of the states. Moreover, open-shell Slater determinants provide a spin-incomplete description of excited states (the initial state in an excited-state XAS calculation), which results in severe spin contamination of all states and may affect the quality of the computed spectra. Hait and Head-Gordon have presented SGM as an alternative general excited-state orbital-optimization method^48^ and applied it to compute XAS spectra of radicals.^49^

Applications of methods containing some empirical component, such as TDDFT, require benchmarking against the spectra computed with a reliable wave-function method, whose accuracy can be systematically assessed. Among various post-HF methods, coupled-cluster (CC) theory yields a hierarchy of size-consistent ansatz for the ground state, with the CC singles and doubles (CCSD) method being the most practical.^50^ CC theory has been extended to excited states via linear response^51–53^ and equation-of-motion for excited states (EOM-EE)^54–57^ formalisms. Both approaches have been adapted to treat core-excited states by using the CVS scheme,^58^ including calculations of transition dipole moments and other properties.^59–65^ The benchmarks illustrate that the CVS-enabled EOM-CC methods describe well the relaxation effects caused by the core hole as well as differential correlation effects. Given their robustness and reliability, the CC-based methods provide high-quality XAS spectra, which can be used to benchmark other methods. Aside from several CCSD investigations,^21,58–60,65–74^ core excitation and ionization energies have also been reported at the CC2 (coupled cluster singles and approximate doubles),^66–68,73,75^ CC3 (coupled cluster singles, doubles and approximate triples),^21,76–78^ CCSDT (coupled cluster singles, doubles and triples),^68,76,79^ CCSDR(3),^66,73,79^ and EOM-CCSD^*^^79^ levels of theory. XAS spectra have also been simulated with a linear-response (LR-)density cumulant theory (DCT),^80^ which is closely related to the LR-CC methods.

The algebraic diagrammatic construction (ADC) approach^81,82^ has also been used to model inner-shell spectroscopy. The second-order variant ADC(2)^83^ yields valence-excitation energies with an accuracy and a computational cost [$O(N^{5})$] similar to CC2,^84^ but within the Hermitian formalism. ADC(2) was extended to core excitations by the CVS scheme.^85,86^ Because ADC(2) is inexpensive and is capable of accounting for dynamic correlation when calculating potential energy surfaces,^87^ it promises to deliver reasonably accurate time-resolved XAS spectra at a low cost at each step of nuclear dynamic simulations. Neville *et al.* simulated TR-XAS spectra with ADC(2)^88–90^ using multireference first-order configuration interaction (MR-FOCI) in their nuclear dynamics simulations. Neville and Schuurman also reported an approach to simulate XAS spectra using electronic wave packet autocorrelation functions based on TD-ADC(2).^91^ An *ad hoc* extension of ADC(2), ADC(2)-x,^92^ is known to give ground-state XAS spectra with relatively high accuracy [better than ADC(2)] employing small basis sets such as 6–31+G,^93^ but the improvement comes with a higher computational cost $\left[O(N^{6})\right]$. List *et al.* have recently used ADC(2)-x, along with restricted active-space second-order perturbation theory (RASPT2), to study competing relaxation pathways in malonaldehyde by TR-XAS simulations.^94^

An important limitation of the single-reference methods (at least those only including singles and double excitations) is that they can reliably treat only singly excited states. While transitions to the singly occupied molecular orbitals (SOMO) result in target states that are formally singly excited from the ground-state reference state, other final states accessible by core excitation from valence-excited states can be dominated by configurations of double or higher excitation character relative to the ground-state reference. Consequently, these states are not well described by conventional response methods such as TDDFT, LR/EOM-CCSD, or ADC(2) (see Fig. 2 in II A).^60,94^ This is the main rational for using MOM within TDDFT. To overcome this problem while retaining a low computational cost, Seidu *et al.*^95^ suggested to combine DFT and multireference configuration interaction (MRCI) with the CVS scheme, which led to the CVS-DFT/MRCI method. The authors demonstrated that the semi-empirical Hamiltonian adjusted to describe the Coulomb and exchange interactions of the valence-excited states^96^ works well for the core-excited states too.

In the context of excited-state nuclear dynamics simulations based on complete active-space SCF (CASSCF) or CAS second-order perturbation theory (CASPT2), popular choices for computing core excitations from a given valence-excited state are restricted active-space SCF (RASSCF)^97,98^ or RASPT2.^99^ Delcey *et al.* have clearly summarized how to apply RASSCF for core excitations.^100^ XAS spectra of valence-excited states computed by RASSCF/RASPT2 have been presented by various authors.^47,101,102^ RASSCF/RASPT2 schemes are sufficiently flexible and even work in the vicinity of conical intersections; they also can tackle different types of excitations, including, for example, those with multiply excited character.^103^ However, the accuracy of these methods depends strongly on an appropriate selection of the active space, which makes their application system specific. In addition, RASSCF simulations might suffer from insufficient description of dynamic correlation, whereas the applicability of RASPT2 may be limited by its computational cost.

Many of the methods mentioned above are available in standard quantum chemistry packages. Hence, the assessment of their performance would help for computational chemists who want to use these methods to analyze the experimental TR-XAS spectra. Since experimental TR-XAS spectra are still relatively scarce, we set out assessing the performance of four selected single-reference methods from the perspective of the three requirements stated above. That is, they should be able to accurately describe the core and valence excitations from the ground state (GS), to give the transition strengths between the core-excited and valence-excited states, and yield the XAS spectra of the valence-excited states over the entire pre-edge region, i.e., describe the spectral features due to the transitions of higher excitation character. More specifically, we extend the use of the MOM approach to the CCSD framework and evaluate its accuracy relative to standard fc-CVS-EOM-EE-CCSD and to MOM-TDDFT. We note that MOM has been used in combination with CCSD to calculate double core excitations.^104^ For selected ground-state XAS simulations, we also consider ADC(2) results.

**FIG. 1.**
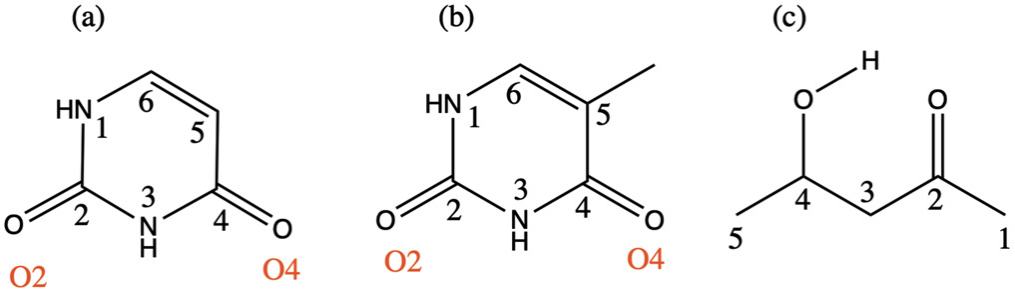
Structures of (a) uracil, (b) thymine, and (c) acetylacetone.

We use the following systems to benchmark the methodology: uracil, thymine, and acetylacetone (Fig. 1). Experimental TR-XAS spectra have not been recorded for uracil yet, but its planar symmetry at the Franck–Condon (FC) geometry and its similarities with thymine make it a computationally attractive model system. Experimental TR-XAS data are available at the O K-edge of thymine and at the C K-edge of acetylacetone.

The paper is organized as follows: First, we describe the methodology and computational details. We then compare the results obtained with the CVS-ADC(2), CVS-EOM-CCSD, and TDDFT methods against the experimental ground-state XAS spectra.^20–22,105^ We also compare the computed valence-excitation energies with UV absorption and electron energy loss spectroscopy (EELS, often called electron impact spectroscopy when it is applied to gas-phase molecules).^106^ We then present the XAS spectra of the valence-excited states obtained with different CCSD-based protocols and compare them with experimental TR-XAS spectra when available.^20–22^ Finally, we evaluate the performance of MOM-TDDFT.

## II. METHODOLOGY

### A. Protocols for computing XAS

**FIG. 2.**
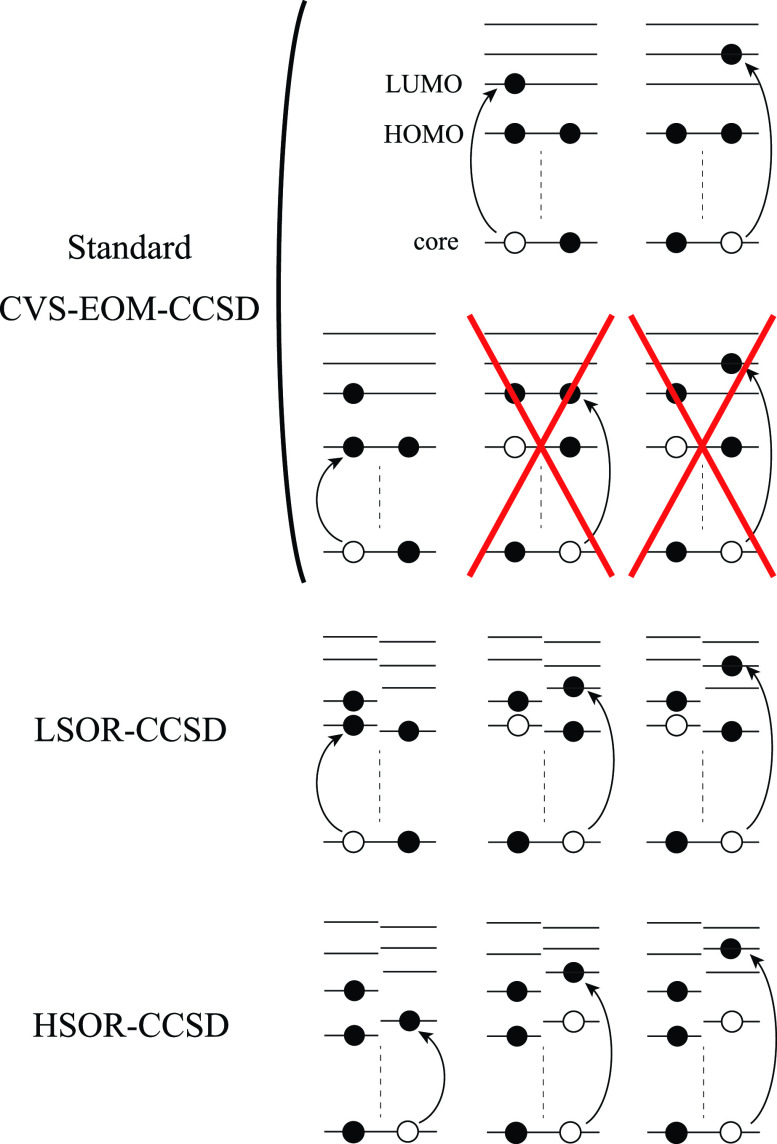
Schematics of the standard CVS-EOM-CCSD, LSOR-CCSD, and HSOR-CCSD protocols. The crossed configurations are formally doubly excited with respect to the ground-state reference.

We calculated the energies and oscillator strengths for core and valence excitations from the ground states by standard LR/EOM methods: ADC(2),^81,82,92^ EOM-EE-CCSD,^50,54–57,107,108^ and TDDFT. In the ADC(2) and CCSD calculations of the valence-excited states, we employ the frozen core (fc) approximation. CVS^58,59,86^ was applied to obtain the core-excited states within all methods. Within the fc-CVS-EOM-EE-CCSD framework,^59^ we explored three different strategies to obtain the excitation energies and oscillator strengths for selected core-valence transitions, as summarized in Fig. 2. In the first one, referred to as standard CVS-EOM-CCSD, we assume that the final core-excited states belong to the set of excited states that can be reached by core excitation from the ground states (see Fig. 2, top panel). Accordingly, we use the HF Slater determinant, representing the ground state ($\left|\Phi_{0}\right\rangle$) as the reference ($\left|\Phi_{\text{ref}}\right\rangle$) for the CCSD calculation; the (initial) valence-excited and (final) core-excited states are then computed with EOM-EE-CCSD and fc-CVS-EOM-EE-CCSD, respectively. The transition energies for core-valence excitations are subsequently computed as the energy differences between the final core states and the initial valence state. The oscillator strengths for the transitions between the two excited states are obtained from the transition moments between the EOM states according to the EOM-CC theory.^50,54,59^ In this approach, both the initial and the final states are spin-pure states. However, the final core-hole states that have multiple excitation character with respect to the ground state are either not accessed or described poorly by this approach (the respective configurations are crossed in Fig. 2).

In the second approach, named high-spin open-shell reference (HSOR) CCSD, we use as a reference for the CCSD calculations a high-spin open-shell HF Slater determinant that has the same electronic configuration as the initial singlet valence-excited state to be probed in the XAS step.^60,64,109^ This approach is based on the assumption that the exchange interactions, which are responsible for the energy gap between singlets and triplets, cancel out in calculations of the transition energies and oscillator strengths. An attractive feature of this approach is that the reference is spin complete (as opposed to a low-spin open-shell determinant of the same occupation) and that the convergence of the SCF procedure is usually robust. A drawback of this approach is the inability to distinguish between the singlet and triplet states with the same electronic configurations.

In the third approach, we use low-spin (M_*s*_ = 0) MOM references for singlet excited states and high-spin (M_*s*_ = 1) MOM references for triplet excited states. We refer to this approach as low-spin open-shell reference (LSOR) CCSD.

In both HSOR-CCSD and LSOR-CCSD, the calculation begins with an SCF optimization targeting the dominant configuration of the initial valence-excited state by means of the MOM algorithm, and the resulting Slater determinant is then used as the reference in the subsequent CCSD calculation. Core-excitation energies and oscillator strengths from the high-spin and the low-spin references are computed with standard CVS-EOM-EE-CCSD. Such MOM-based CCSD calculations can describe all target core-hole states, provided that they have singly excited character with respect to the chosen reference. Furthermore, in principle, initial valence-excited states of different spin symmetries can be selected. However, in calculations using low-spin open-shell references (LSOR-CCSD states), variational collapse might occur. Moreover, the LSOR-CCSD treatment of singlet excited states suffers from spin contamination as the underlying open-shell reference is not spin complete (the well known issue of spin-completeness in calculations using open-shell references is discussed in detail in recent review articles.^110,111^).

We note that the HSOR-CCSD ansatz for a spin-singlet excited state is identical to the LSOR-CCSD ansatz of a (M_*s*_ = 1) spin-triplet state having the same electronic configuration as the spin-singlet excited state (see Fig. 2).

In addition to the three CCSD-based protocols described above, we also considered MOM-TDDFT, which is often used for simulation of the time-resolved near-edge x-ray absorption fine structure (TR-NEXAFS) spectra.^20,22,47^ We employed the B3LYP xc-functional,^44^ as in Refs. 20, 22, and 47.

### B. Computational details

The equilibrium geometry of uracil was optimized at the MP2/cc-pVTZ level. The equilibrium geometries of thymine and acetylacetone were taken from the literature;^21,61^ they were optimized at the CCSD(T)/aug-cc-pVDZ and CCSD/aug-cc-pVDZ level, respectively. These structures represent the molecules at the FC points. The structures of the T_1_($\pi \pi^{*}$) and S_1_(n$\pi^{*}$) states of acetylacetone, and of the S_1_(n$\pi^{*}$) state of thymine were optimized at the EOM-EE-CCSD/aug-cc-pVDZ level.^61^

We calculated near-edge x-ray absorption fine structure (NEXAFS) of the ground state of all three molecules using CVS-ADC(2), CVS-EOM-CCSD, and TDDFT/B3LYP. The excitation energies of the valence-excited states were calculated with ADC(2), EOM-EE-CCSD, and TDDFT/B3LYP. The XAS spectra of the T_1_($\pi \pi^{*}$), T_2_(n$\pi^{*}$), S_1_(n$\pi^{*}$), and S_2_($\pi \pi^{*}$) states of uracil were calculated at the FC geometry. We used the FC geometry for all states in order to make a coherent comparison of the MOM-based CCSD methods with the standard CCSD method and to ensure that the final core-excited states are the same in the ground state XAS and transient state XAS calculations using standard CCSD. The spectra of thymine in the S_1_(n$\pi^{*}$) state were calculated at the potential energy minimum of the S_1_(n$\pi^{*}$) state. The spectra of acetylacetone in the T_1_($\pi \pi^{*}$) and S_2_($\pi \pi^{*}$) states were calculated at the potential energy minima of the T_1_($\pi \pi^{*}$) and S_1_(n$\pi^{*}$) states, respectively. Our choice of geometries for acetylacetone is based on the fact that the S_2_($\pi \pi^{*}$)-state spectra were measured during wave packet propagation from the S_2_($\pi \pi^{*}$) minimum (planar) toward the S_1_(n$\pi^{*}$) minimum (distorted), and the ensemble was in equilibrium when the T_1_($\pi \pi^{*}$)-state spectra were measured.^22^

The XAS spectra of the valence-excited states were computed with CVS-EOM-CCSD, HSOR-CCSD, and LSOR-CCSD. Pople's 6–311++G^**^ basis set was used throughout. In each spectrum, the oscillator strengths were convoluted with a Lorentzian function (empirically chosen FWHM = 0.4 eV,^60^ unless otherwise specified). We used the natural transition orbitals (NTOs)^37,112–119^ to determine the character of the excited states.

All calculations were carried out with the Q-Chem 5.3 electronic structure package.^120^ The initial guesses [HOMO(*β*)]^1^[LUMO(*α*)]^1^ and [HOMO(*α*)]^1^[LUMO(*α*)]^1^ were used in MOM-SCF for the spin-singlet and triplet states dominated by (HOMO)^1^(LUMO)^1^ configuration, respectively. The SOMOs of the initial guess in a MOM-SCF procedure are the canonical orbitals (or the Kohn–Sham orbitals) which resemble the hole and particle NTO of the transition from the ground state to the valence-excited state. One should pay attention to the order of the orbitals obtained in the ground-state SCF, especially when the basis set has diffuse functions. In LSOR-CCSD calculations, the SCF convergence threshold had to be set to $10^{-9}$ Hartree. To ensure convergence to the dominant electronic configuration of the desired electronic state, we used the initial MOM (IMOM) algorithm^121^ instead of regular MOM; this is important for cases when the desired state belongs to the same irreducible representation as the ground state.

## III. RESULTS AND DISCUSSION

### A. Ground-state NEXAFS

**FIG. 3.**
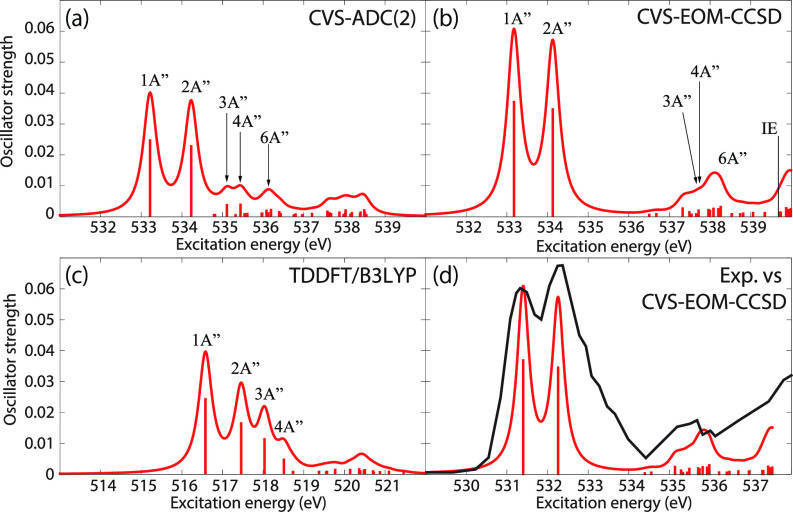
Uracil. Ground-state NEXAFS at the oxygen K-edge calculated with (a) ADC(2); (b) CVS-EOM-CCSD; (c) TDDFT/B3LYP. The calculated IEs are 539.68 and 539.86 eV (fc-CVS-EOM-IP-CCSD/6-311++G^**^). In panel (d), the computed spectrum of (b) is shifted by −1.8 eV and superposed with the experimental spectrum^105^ (black curve). Basis set: 6-311++G^**^.

Figure 3 shows the O K-edge NEXAFS spectra of uracil in the ground state computed by CVS-EOM-CCSD, CVS-ADC(2), and TDDFT/B3LYP. Table I shows NTOs of the core-excited states calculated at the CVS-EOM-CCSD/6–311++G^**^ level, where *σ_K_* are the singular values for a given NTO pair (their renormalized squares give the weights of the respective configurations in the transition).^37,112–119^ The NTOs for the other two methods are collected in the supplementary material. Panel (d) of Fig. 3 shows the experimental spectrum (digitized from Ref. 105). The experimental spectrum has two main peaks at 531.3 and 532.2 eV, assigned to core excitations to the $\pi^{*}$ orbitals from O4 and O2, respectively. Beyond these peaks, the intensity remains low up to 534.4 eV. The next notable spectral feature, attributed to Rydberg excitations, emerges at around 535.7 eV, just before the first core-ionization onset (indicated as IE). The separation of ∼0.9 eV between the two main peaks is reproduced at all three levels of theory. The NTO analysis at the CCSD level (cf. Table I) confirms that the excitation to the 6A″ state has Rydberg character and, after the uniform shift, the peak assigned to this excitation falls in the Rydberg region of the experimental spectrum. ADC(2) also yields a 6A″ transition of Rydberg character, but it is significantly red-shifted relative to the experiment. No Rydberg transitions are found at the TDDFT level. Only CVS-EOM-CCSD reproduces the separation between the 1A″ and the 6A″ peaks with reasonable accuracy, 4.91 eV vs 4.4 eV in the experimental spectrum. The shoulder structure of the experimental spectrum in the region between 532.2 and 534.4 eV is attributed to vibrational excitations or shakeup transitions.^18,122^

**TABLE I.** Uracil. CVS-EOM-CCSD/6-311++G^**^ energies, strengths, and NTOs of the O_1*s*_ core excitations from the ground state at the FC geometry (NTO isosurface is 0.04 for the Rydberg transition and 0.05 for the rest).

Final state	$E^{\text{ex}}$ (eV)	Osc. strength	Hole	$\sigma_{K}^{2}$	Particle
1A″	533.17	0.036 7	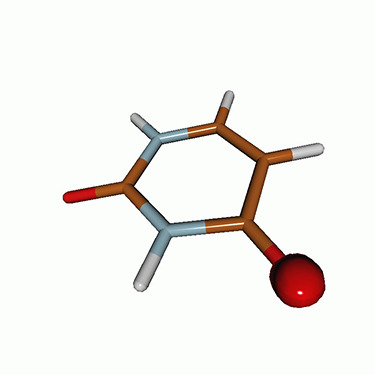	0.78	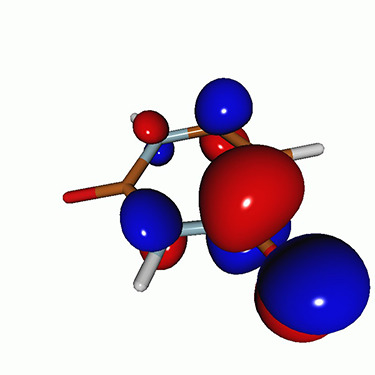
2A″	534.13	0.034 3	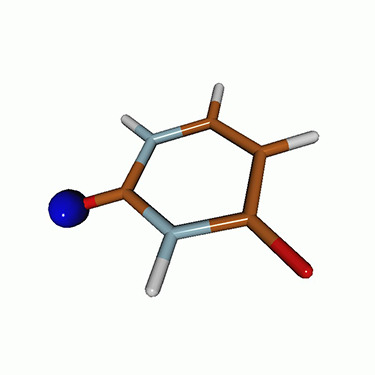	0.79	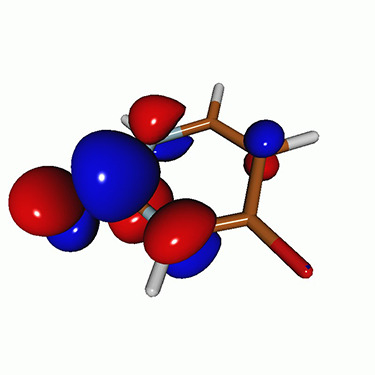
3A″	537.55	0.000 3	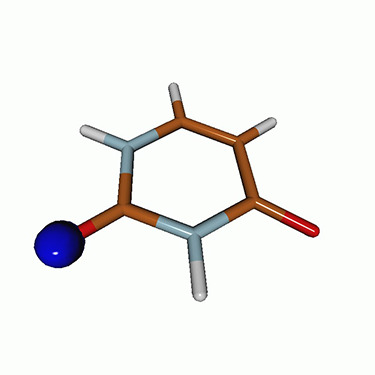	0.76	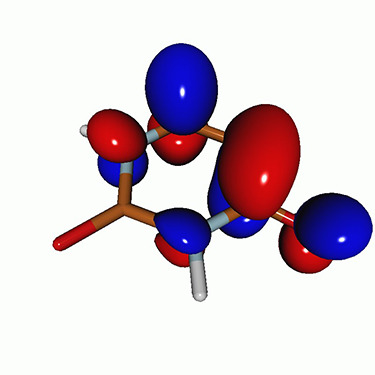
4A″	537.66	0.000 4	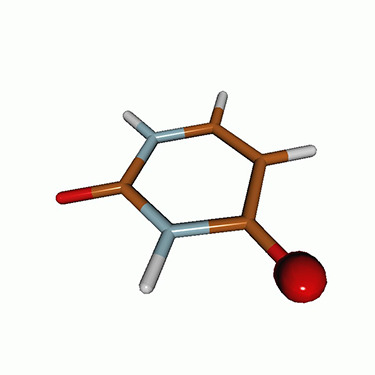	0.78	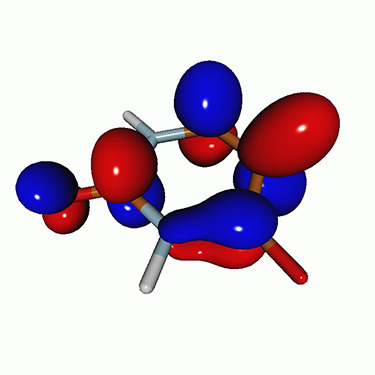
6A″	538.08	0.002 2	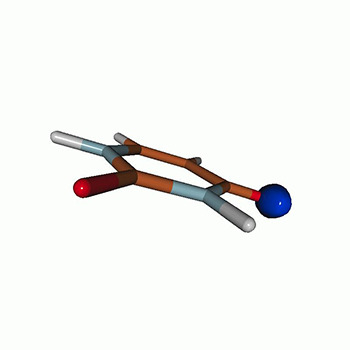	0.82	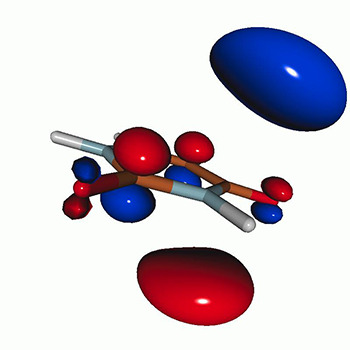

**FIG. 4.**
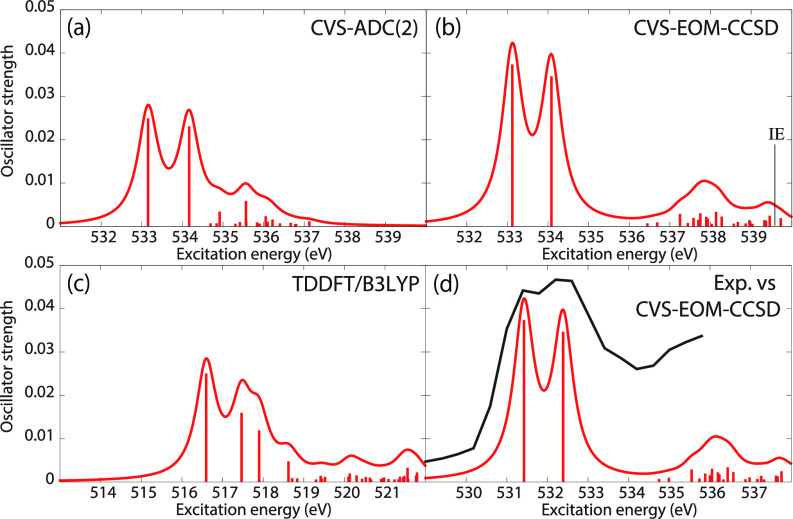
Thymine. Ground-state oxygen K-edge NEXAFS calculated with (a) ADC(2), (b) CVS-EOM-CCSD, (c) TDDFT/B3LYP. The computed ionization energies (IEs) are 539.67 and 539.73 eV (fc-CVS-EOM-IP-CCSD). In panel (d), the CVS-EOM-CCSD spectrum of (b) is shifted by −1.7 eV and superposed with the experimental one^21^ (black curve). Basis set: 6-311++G^**^. FWHM of the Lorentzian convolution function is 0.6 eV.

Figure 4 shows the ground-state NEXAFS spectra of thymine at the O K-edge. For construction of the theoretical absorption spectra, we used FWHM of 0.6 eV for the Lorentzian convolution function. Panel (d) shows the experimental spectrum (digitized from Ref. 21). Both the experimental and calculated spectra exhibit fine structures, similar to those of uracil. Indeed, the first and second peaks at 531.4 and 532.2 eV of the experimental spectrum were assigned to O_1*s*_-hole states having the same electronic configuration characters as the two lowest-lying O_1*s*_-hole states of uracil. The NTOs of thymine can be found in the supplementary material. Again, only CVS-EOM-CCSD reproduces reasonably well the Rydberg region after 534 eV. The separation of the two main peaks is well reproduced at all three levels of theory.

**FIG. 5.**
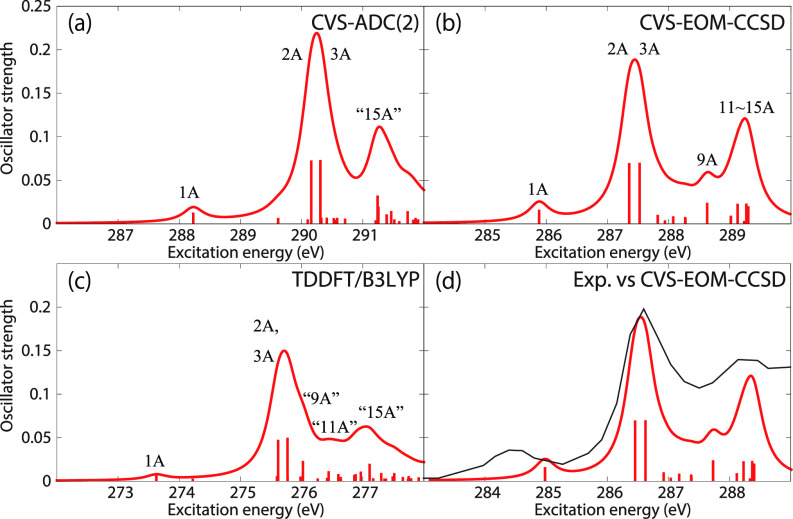
Acetylacetone. Ground-state NEXAFS at carbon K-edge calculated with (a) ADC(2); (b) CVS-EOM-CCSD; (c) TDDFT/B3LYP. The ionization energies (IEs) are 291.12, 291.88, 292.11, 294.10, and 294.56 eV (fc-CVS-EOM-IP-CCSD). In panel (d), the computational result of (b) is shifted by −0.9 eV and superposed with the experimental spectrum^22^ (black curve). Basis set: 6-311++G^**^.

Figure 5 shows the C K-edge ground-state NEXAFS spectra of acetylacetone; the NTOs of the core excitations obtained at the CVS-EOM-CCSD/6–311++G^**^ level are collected in Table II. The experimental spectrum, plotted in panel (d) of Fig. 5, was digitized from Ref. 22. Table II shows that the first three core excitations are dominated by the transitions to the LUMO from the 1*s* orbitals of the carbon atoms C2, C3, and C4. Transition from the central carbon atom, C3, appears as the first relatively weak peak at 284.4 eV. We note that acetylacetone may exhibit keto–enol tautomerism. In the keto form, atoms C2 and C4 are equivalent. Therefore, transitions from these carbon atoms appear as quasi-degenerate main peaks at ≈286.6 eV. The region around 288.2 eV is attributed to Rydberg transitions. The ∼2 eV separation between the first peak and the main peak due to the two quasi-degenerate transitions is well reproduced by ADC(2) and TDDFT/B3LYP, and slightly underestimated by CVS-EOM-CCSD (1.6 eV). On the other hand, the separation of ∼1.6 eV between the main peak and the Rydberg resonance region is well reproduced only by CVS-EOM-CCSD.

**TABLE II.** Acetylacetone. CVS-EOM-CCSD/6-311++G^**^ NTOs of the C_1*s*_ core excitations from the ground state at the FC geometry (NTO isosurface is 0.03 for the Rydberg transition and 0.05 for the rest).

Final state	$E^{\text{ex}}$ (eV)	Osc. strength	Hole	$\sigma_{K}^{2}$	Particle
1A	285.88	0.013 3	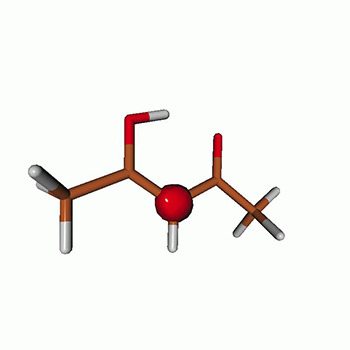	0.76	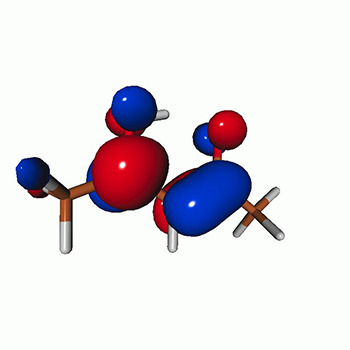
2A	287.36	0.067 1	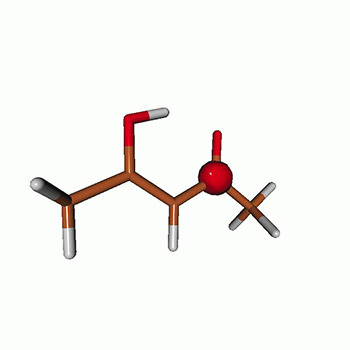	0.82	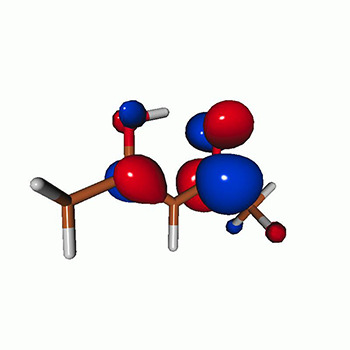
3A	287.53	0.067 3	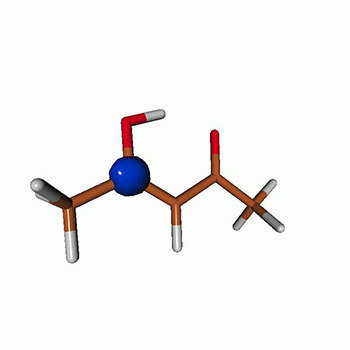	0.81	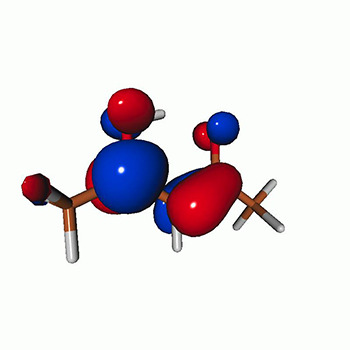
9A	288.63	0.021 3	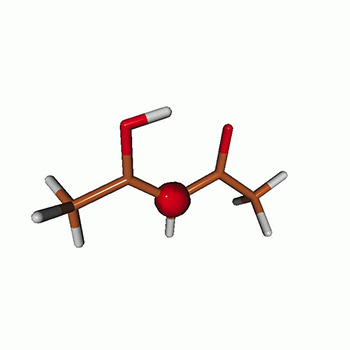	0.79	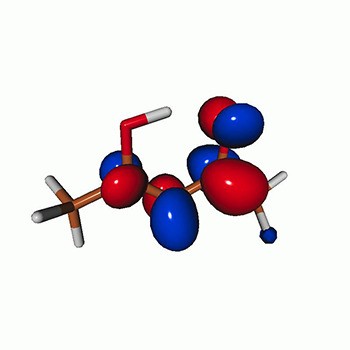
11A	289.13	0.020 2	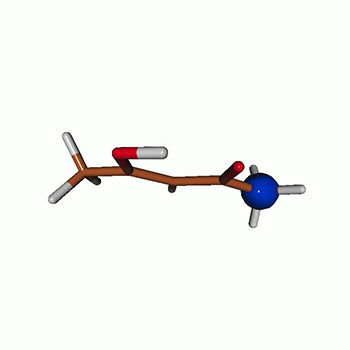	0.82	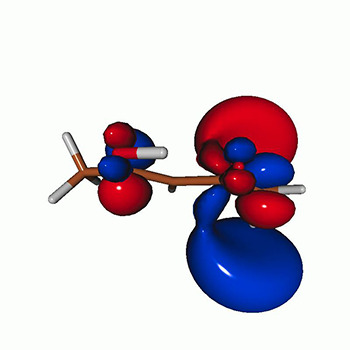
13A	289.27	0.020 5	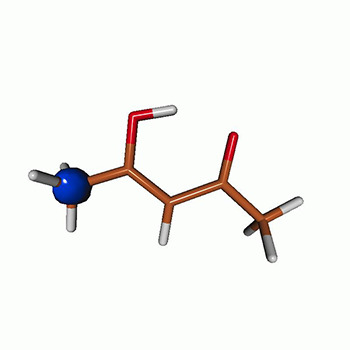	0.83	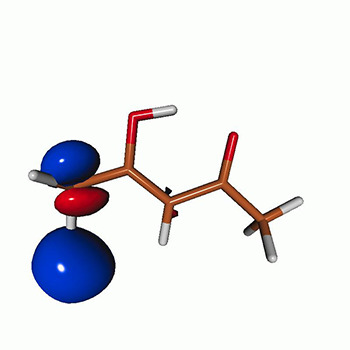
14A	289.28	0.017 5	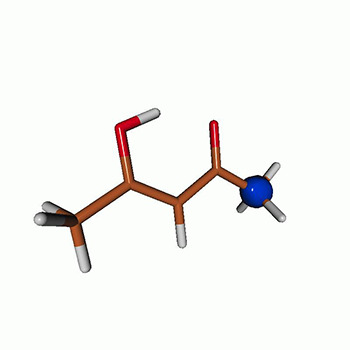	0.82	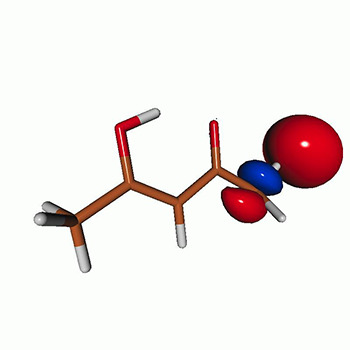
15A	289.30	0.017 4	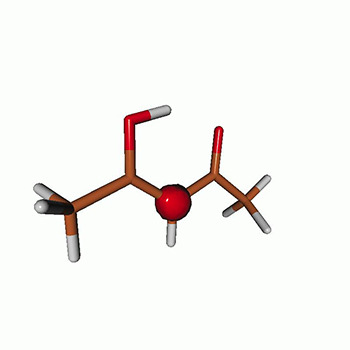	0.81	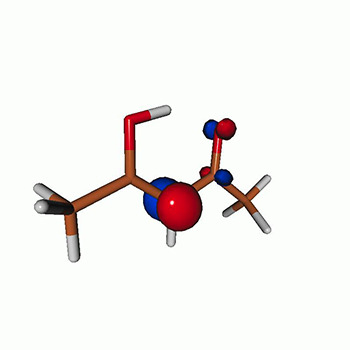

The results for the three considered molecules illustrate that CVS-EOM-CCSD describes well the entire pre-edge region of the NEXAFS spectrum. CVS-ADC(2) and TDDFT/B3LYP describe well the core excitations to the LUMO and LUMO+1 (apart from a systematic shift), but generally fail to describe the transitions at higher excitation energies.

### B. Valence-excited states

Table III shows the excitation energies of the two lowest triplet states, the three lowest singlet states, plus the S_5_($\pi \pi^{*}$) state of uracil, calculated at the FC geometry, along with the values derived from the EELS^123^ and UV absorption experiments.^124^ The EOM-EE-CCSD/6–311++G^**^ NTOs are collected in Table IV, and the NTOs for other methods are given in the supplementary material. We refer to Ref. 125 for an extensive benchmark study of the one-photon absorption and excited-state absorption of uracil.

**TABLE III.** Uracil. Excitation energies (eV) at the FC geometry and comparison with experimental values from EELS^123^ and UV absorption spectroscopy.^124^

	ADC(2)	ADC(2)-x	EOM-CCSD	TDDFT	EELS	UV
T_1_($\pi \pi^{*}$)	3.91	3.36	3.84	3.43	3.75	
T_2_(n$\pi^{*}$)	4.47	3.79	4.88	4.27	4.76	
S_1_(n$\pi^{*}$)	4.68	3.93	5.15	4.65	5.2	
S_2_($\pi \pi^{*}$)	5.40	4.70	5.68	5.19	5.5	5.08
S_3_(πRyd)	5.97	5.39	6.07	5.70	…	
S_5_($\pi \pi^{*}$)	6.26	5.32	6.74	5.90	6.54	6.02

**TABLE IV.** Uracil. EOM-EE-CCSD/6-311++G^**^ NTOs for the transitions from the ground state to the lowest valence-excited states at the FC geometry (NTO isosurface is 0.05).

Final state	$E^{\text{ex}}$ (eV)	Osc. strength	Hole	$\sigma_{K}^{2}$	Particle
T1($A^{\prime },\;\pi \pi^{*}$)	3.84	…	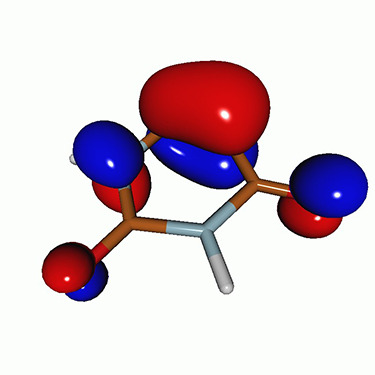	0.82	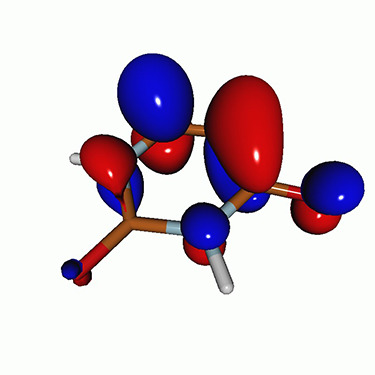
T2(A^″^, n$\pi^{*}$)	4.88	…	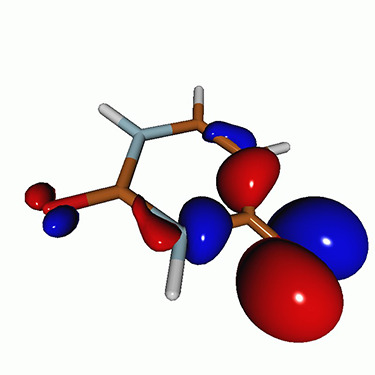	0.82	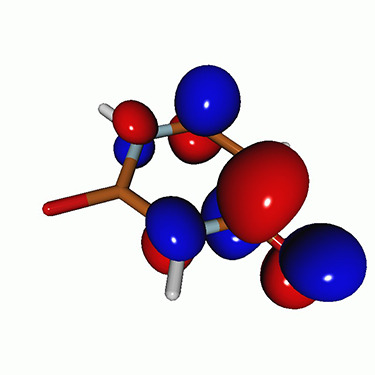
S1(A^″^, n$\pi^{*}$)	5.15	0.000 0	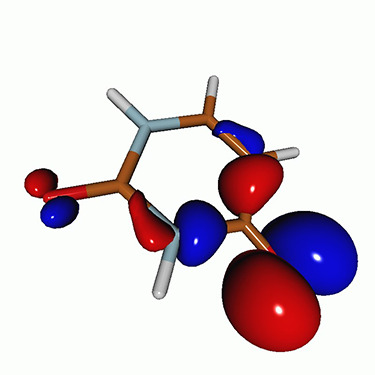	0.81	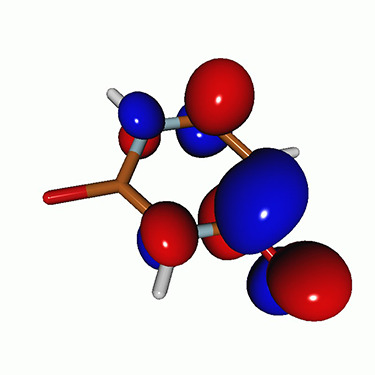
S2(A′, $\pi \pi^{*}$)	5.68	0.238 6	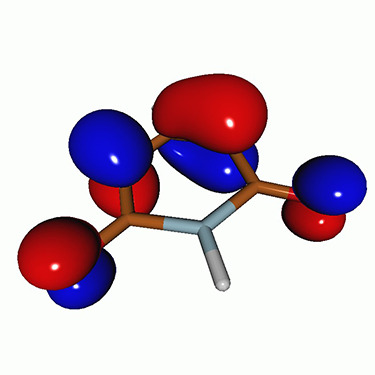	0.75	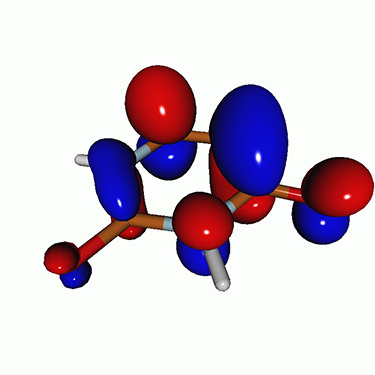
S3($A^{\prime \prime },\;\pi \text{Ryd}$)	6.07	0.0027	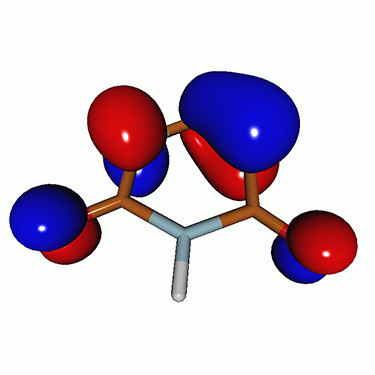	0.85	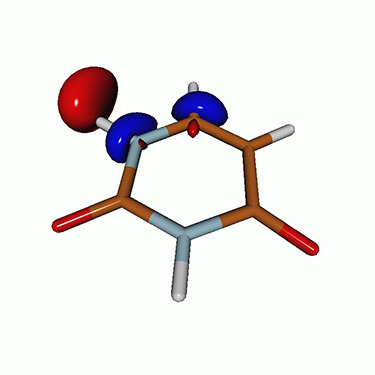
S5(A′, $\pi \pi^{*}$)	6.74	0.0573	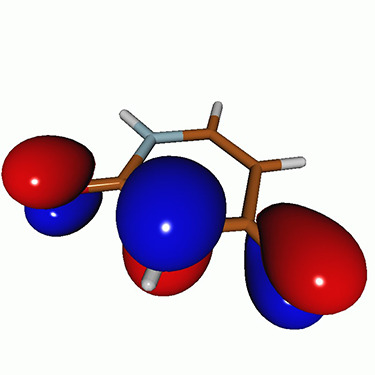	0.73	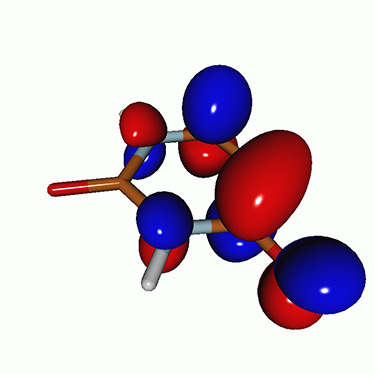

In EELS, the excited states are probed by measuring the kinetic energy change of a beam of electrons after inelastic collision with the probed molecular sample.^106^ In the limit of high incident energy or small scattering angle, the transition amplitude takes a dipole form and the selection rules are same as those of UV-Vis absorption. Otherwise, the selection rules are different and optically dark states can be detected. Furthermore, spin–orbit coupling enables excitation into triplet states. Assignment of the EELS spectral signatures is based on theoretical calculations. Note that excitation energies obtained with EELS may be blue-shifted compared to those from UV-Vis absorption due to momentum transfer between the probing electrons and the probed molecule.

EOM-EE-CCSD excitation energies for all valence states of uracil agree well with the experimental values from EELS. Both the EOM-EE-CCSD and EELS values slightly overestimate the UV-Vis results. For the two triplet states and the S_1_(A″, n$\pi^{*}$) and S_2_($A^{\prime },\;\pi \pi^{*}$) states, ADC(2) also gives fairly accurate excitation energies. ADC(2)-x, on the other hand, seems unbalanced for the valence excitations (regardless of the basis set). The TDDFT/B3LYP excitation energies are red-shifted with respect to the EELS values, but the energy differences between the T_1_(A$\prime ,\;\pi \pi^{*}$), T_2_(A″, n$\pi^{*}$), S_1_(A″, n$\pi^{*}$), and S_2_($A^{\prime },\;\pi \pi^{*}$) states are in reasonable agreement with the corresponding experimentally derived values.

Table V shows the excitation energies of the five lowest triplet and singlet states of thymine, along with the experimental values obtained by EELS.^126^ We did not find literature data for the UV absorption of thymine in the gas phase. The energetic order is based on EOM-EE-CCSD. Here, we reassign the peaks of the EELS spectra^126^ on the basis of the following considerations: (*i*) optically bright transitions also exhibit strong peaks in the EELS spectra; (*ii*) the excitation energy of a triplet state is lower than the excitation energy of the singlet state with the same electronic configuration; (*iii*) the strengths of the transitions to triplet states are smaller than the strengths of the transitions to singlet states; (*iv*) among the excitations enabled by spin–orbit coupling, $\pi \to \pi^{*}$ transitions have relatively large transition moments.

**TABLE V.** Thymine. Excitation energies (eV) at the FC geometry compared with the experimental values from EELS.^126^ The oscillator strengths are from EOM-EE-CCSD and used for the re-assignment.

	ADC(2)	EOM-CCSD	TDDFT	EELS	Osc. strength
T_1_($\pi \pi^{*}$)	3.70	3.63	3.19	3.66	…
T_2_(n$\pi^{*}$)	4.39	4.81	4.25	4.20	…
S_1_(n$\pi^{*}$)	4.60	5.08	4.64	4.61	0.000 0
S_2_($\pi \pi^{*}$)	5.18	5.48	4.90	4.96	0.228 9
T_3_($\pi \pi^{*}$)	5.27	5.32	4.61	5.41	….
T_4_(πRyd)	5.66	5.76	5.39	…	…
S_3_(πRyd)	5.71	5.82	5.46	…	0.000 5
T_5_($\pi \pi^{*}$)	5.87	5.91	5.10	5.75	…
S_4_(n$\pi^{*}$)	5.95	6.45	5.72	5.96	0.000 0
S_5_($\pi \pi^{*}$)	6.15	6.63	5.87	6.17	0.067 9

Except for T_1_($\pi \pi^{*}$), the ADC(2) excitation energies are red-shifted relative to EOM-CCSD. Hence, the ADC(2) excitation energies of the states considered here are closest, in absolute values, to the experimental values from Table V. However, the energy differences between the singlet states (S_1_, S_2_, S_4_, and S_5_) are much better reproduced by EOM-CCSD. TDDFT/B3LYP accurately reproduces the excitation energies of the T_2_(n$\pi^{*}$), S_1_(n$\pi^{*}$), and S_2_($\pi \pi^{*}$) states.

Table VI shows the excitation energies of the two lowest triplet and singlet states, and the lowest Rydberg states of acetylacetone, along with the experimental values obtained from EELS^127^ and UV absorption^128^ (the exact state ordering of states in the singlet Rydberg manifold is unknown). Table VII shows the NTOs obtained at the EOM-EE-CCSD/6–311++G^**^ level. Remarkably, for this molecule the excitation energies from EELS agree well with those from UV absorption. Note that the EELS spectra of acetylacetone were recorded with incident electron energies of 25 and 100 eV,^127^ whereas those for uracil^123^ were obtained with 0–8.0 eV. The higher incident electron energies reduce the effective acceptance angle of the electrons, which may hinder the detection of electrons that have undergone momentum transfer. The transitions to the T_1_($\pi \pi^{*}$) and T_2_(n$\pi^{*}$) states appeared only with the 25 eV incident electron energy and a scattering angle of 90° (see Fig. 3 of Ref. 127). The peaks were broad and, furthermore, an order of magnitude less intense than the $S_{0}\to$ S_2_($\pi \pi^{*}$) transition. Consequently, it is difficult to resolve the excitation energies of T_1_($\pi \pi^{*}$) and T_2_(n$\pi^{*}$). ADC(2) yields the best match with the experimental results for acetylacetone.

**TABLE VI.** Acetylacetone. Excitation energies (eV) at the FC geometry compared with the values obtained in EELS^127^ and UV absorption spectroscopy.^128^

	ADC(2)	ADC(2)-x	EOM-CCSD	TDDFT	EELS	UV
T_1_($\pi \pi^{*}$)	3.76	3.16	3.69	3.23	3.57?	…
T_2_(n$\pi^{*}$)	3.79	3.13	4.11	3.75	?	…
S_1_(n$\pi^{*}$)	4.03	3.29	4.39	4.18	4.04	4.2
S_2_($\pi \pi^{*}$)	4.96	4.28	5.24	5.08	4.70	4.72
T${}_{3}(\pi \text{Ryd})$	5.91	5.45	6.02	5.66	5.52	…
S${}_{3?}(\pi \text{Ryd})$	5.98	5.53	6.13	5.72	5.84	5.85
S${}_{5?}(\pi \text{Ryd})$	6.87	6.30	7.06	6.64	6.52	6.61

**TABLE VII.** Acetylacetone. EOM-EE-CCSD/6-311++G^**^ NTOs of the excitations from the ground state to the lowest-lying valence-excited states at the FC geometry (NTO isosurface is 0.03 for the Rydberg transitions and 0.05 for the rest).

Final state	$E^{\text{ex}}$ (eV)	Osc. strength	Hole	$\sigma_{K}^{2}$	Particle
T_1_(A′, $\pi \pi^{*}$)	3.69	…	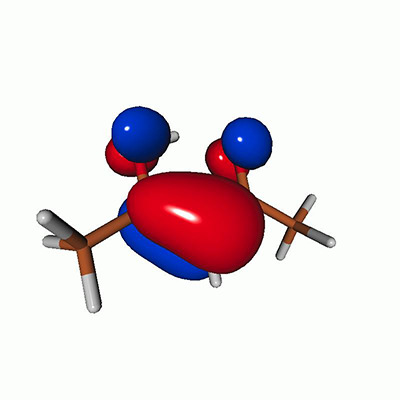	0.82	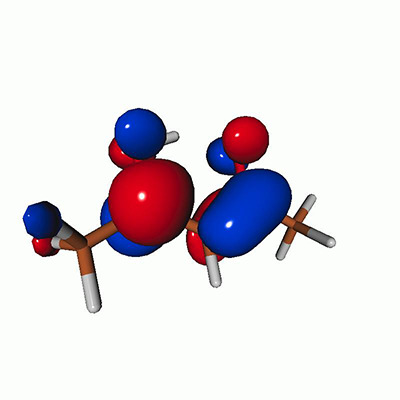
T_2_(A″, n$\pi^{*}$)	4.11	…	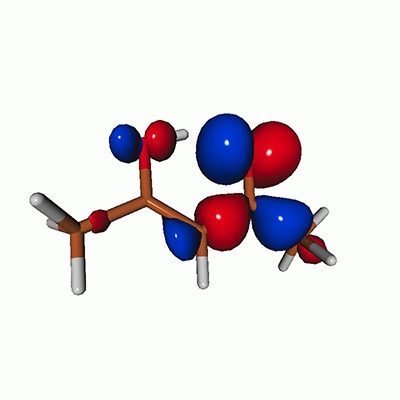	0.82	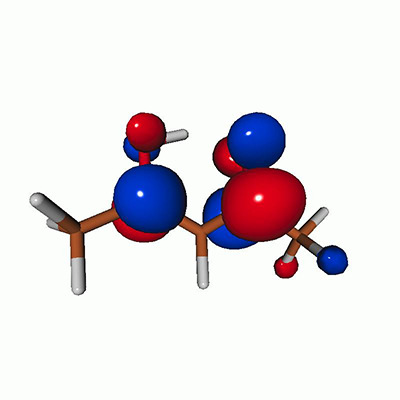
S_1_(A″, n$\pi^{*}$)	4.39	0.000 6	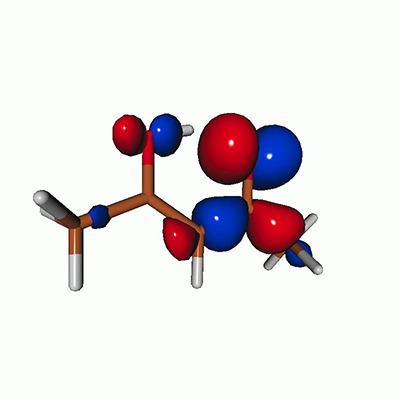	0.81	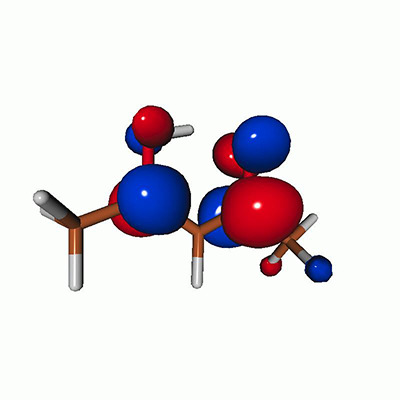
S_2_(A′, $\pi \pi^{*}$)	5.24	0.329 9	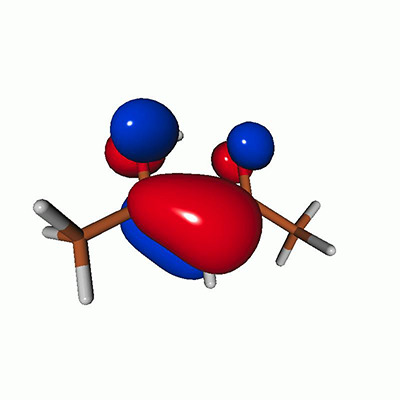	0.77	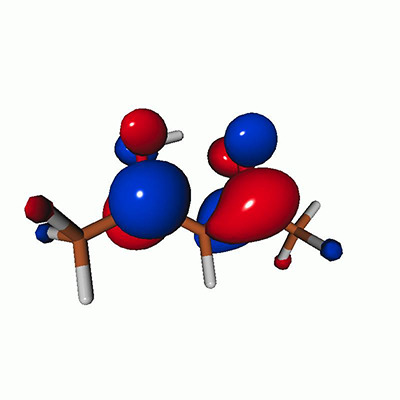
T${}_{3}[\pi \text{Ryd}(s)]$	6.02	…	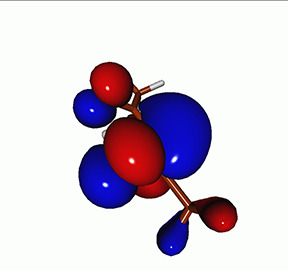	0.86	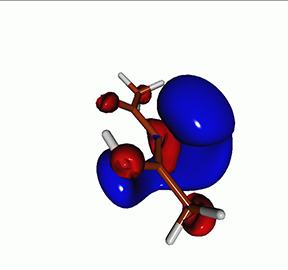
S${}_{3?}[\pi \text{Ryd}(s)]$	6.13	0.007 2	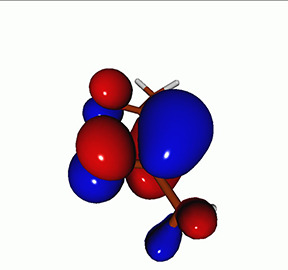	0.86	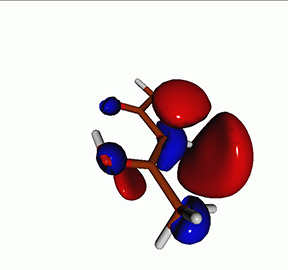
S${}_{5?}[\pi \text{Ryd}(p)]$	7.06	0.057 1	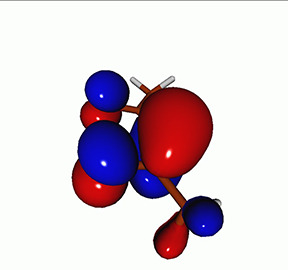	0.85	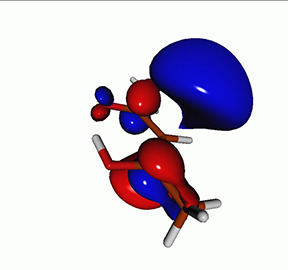

These results indicate that the excitation energies of the valence-excited states computed by EOM-EE-CCSD, ADC(2), and TDDFT/B3LYP are equally (in)accurate. Which method yields the best match with experiment depends on the molecule.

### C. Core excitations from the valence-excited states

In Secs. III A and III B, we analyzed two of our three desiderata for a good electronic structure method for TR-XAS—that is, the ability to yield accurate results for ground-state XAS as well as for the valence-excited states involved in the dynamics. In this subsection, we focus on the remaining item, i.e., the ability to yield accurate XAS of valence-excited states.

For uracil, we confirmed that EOM-CCSD and CVS-EOM-CCSD yield fairly accurate results for the valence-excited T_1_($\pi \pi^{*}$), T_2_($n\pi^{*}$), S_1_($n\pi^{*}$), and S_2_($\pi \pi^{*}$) states and for the (final) singlet (O_1*s*_) core-excited states at the FC geometry, respectively. It is thus reasonable to consider the oxygen K-edge XAS spectra of the S_1_($n\pi^{*}$) and S_2_($\pi \pi^{*}$) states of uracil obtained from CVS-EOM-CCSD as our reference, even though CVS-EOM-CCSD only yields the peaks of the core-to-SOMO transitions.

**FIG. 6.**
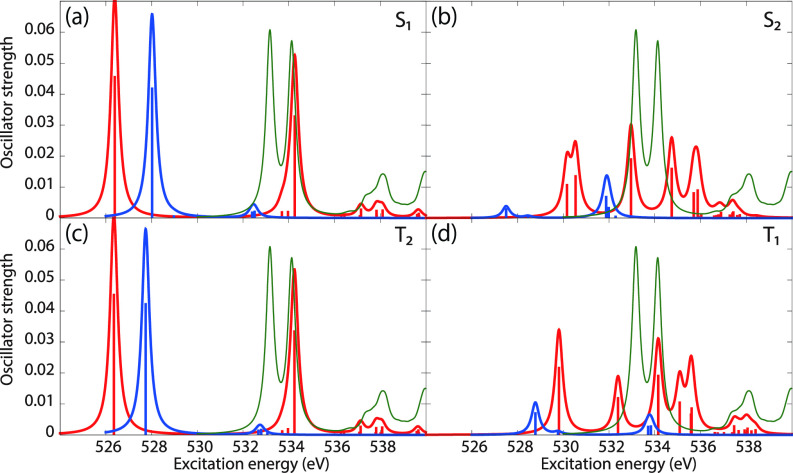
Uracil. Oxygen K-edge NEXAFS of the four lowest-lying valence states: (a) S_1_($n\pi^{*}$); (b) S_2_($\pi \pi^{*}$); (c) T_2_($n\pi^{*}$); and (d) T_1_($\pi \pi^{*}$)]. The blue and red curves correspond to the CVS-EOM-CCSD and LSOR-CCSD results, respectively. Note that the HSOR spectra for S_1_ and S_2_ are identical to the LSOR-CCSD spectra for T_2_ and T_1_. Basis set: 6-311++G^**^. FC geometry. The ground state XAS (green curve) is included for comparison.

Figure 6 shows the oxygen K-edge XAS of uracil in the (a) S_1_($n\pi^{*}$), (b) S_2_($\pi \pi^{*}$), (c) T_2_($n\pi^{*}$), and (d) T_1_($\pi \pi^{*}$) states, calculated using CVS-EOM-CCSD (blue curve) and LSOR-CCSD (red curve) at the FC geometry. Note that the HSOR-CCSD spectra of S_1_($n\pi^{*}$) and S_2_($\pi \pi^{*}$) are identical to the LSOR-CCSD spectra for the T_2_($n\pi^{*}$) and T_1_($\pi \pi^{*}$) states, respectively, because their orbital electronic configuration are the same, see Table IV. The ground-state spectrum (green curve) is included in all panels for comparison. The LSOR-CCSD NTOs of the transitions underlying the peaks in the S_1_($n\pi^{*}$), S_2_($\pi \pi^{*}$) and T_1_($\pi \pi^{*}$) spectra are given in Tables VIII–X, respectively.

**TABLE VIII.** Uracil. LSOR-CCSD/6-311++G^**^ NTOs of the O_1*s*_ core excitations from the S_1_ (n$\pi^{*}$) state at the FC geometry (NTO isosurface value is 0.05).

$E^{\text{ex}}$ (eV)	Osc. strength	Spin	Hole	$\sigma_{K}^{2}$	Particle
526.39	0.045 1	*α*	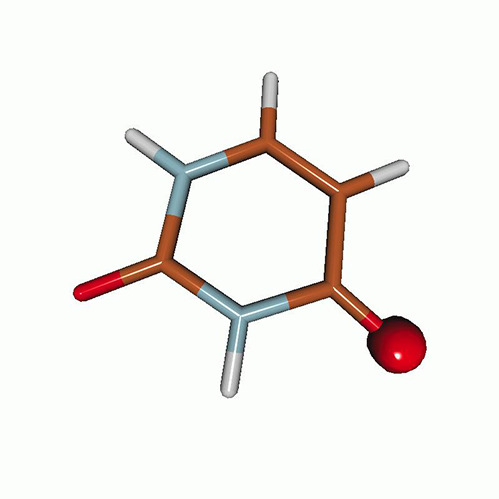	0.86	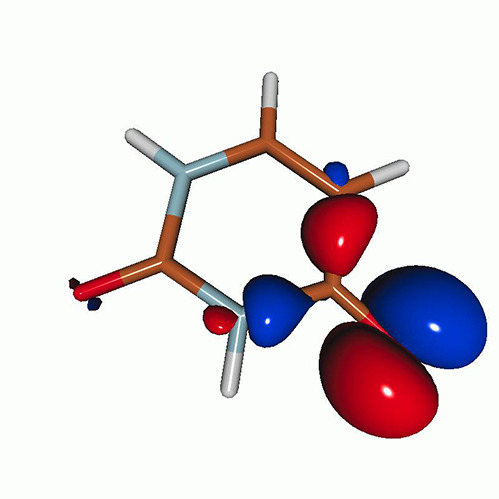
534.26	0.032 3	*α*	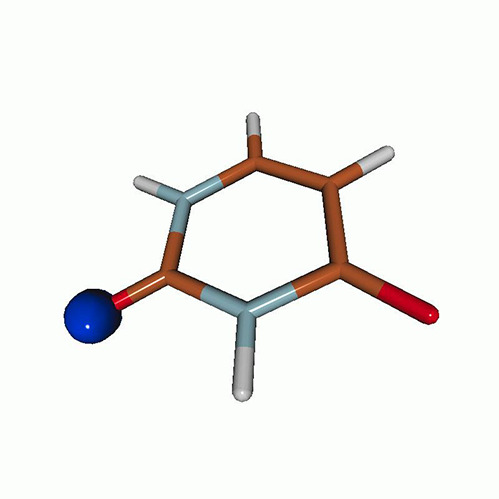	0.56	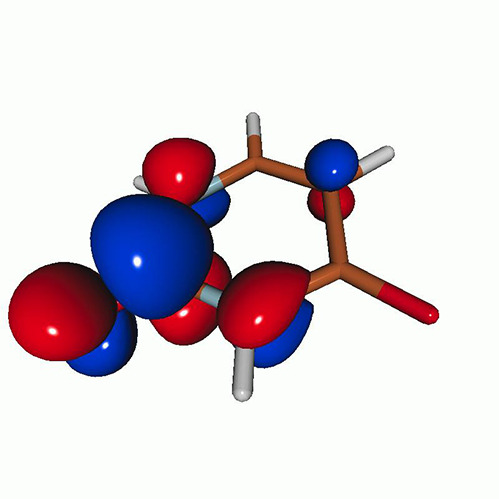
		*β*	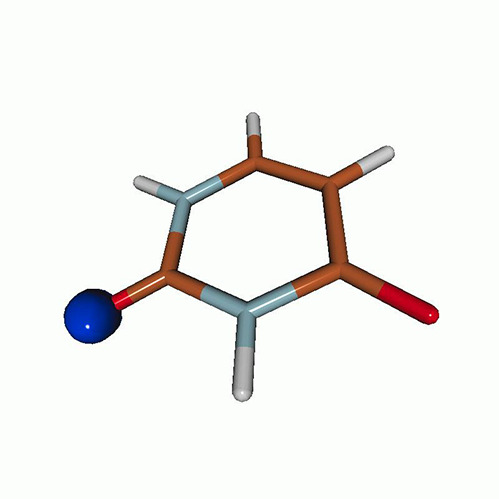	0.23	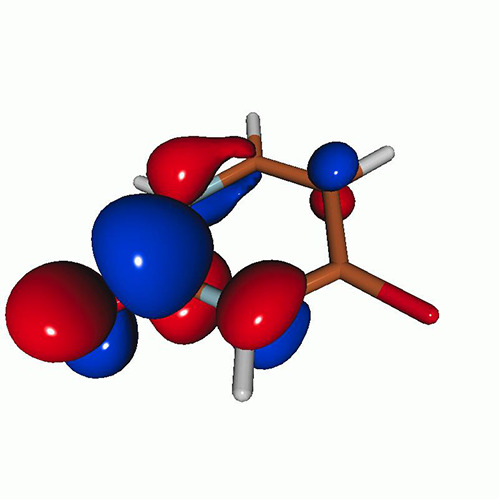

**TABLE IX.** Uracil. LSOR-CCSD/6-311++G^**^ NTOs of the O_1*s*_ core excitations from the S_2_($\pi \pi^{*}$) state at the FC geometry (NTO isosurface value is 0.05).

$E^{\text{ex}}$ (eV)	Osc. strength	Spin	Hole	$\sigma_{K}^{2}$	Particle
530.16	0.010 2	*α*	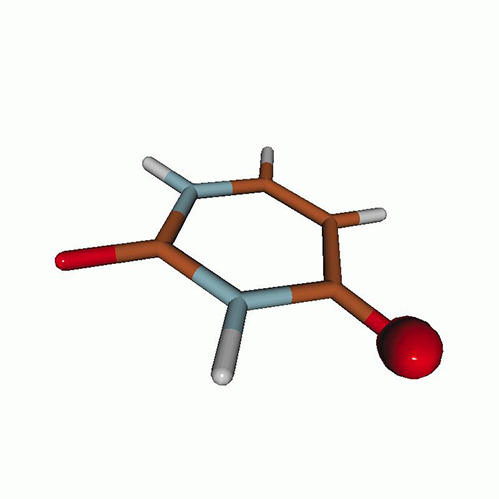	0.68	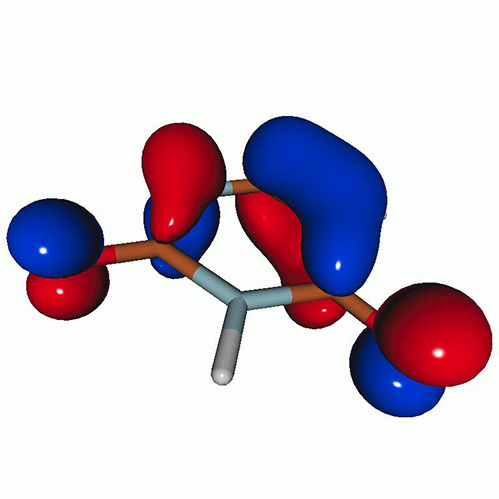
530.54	0.013 1	*α*	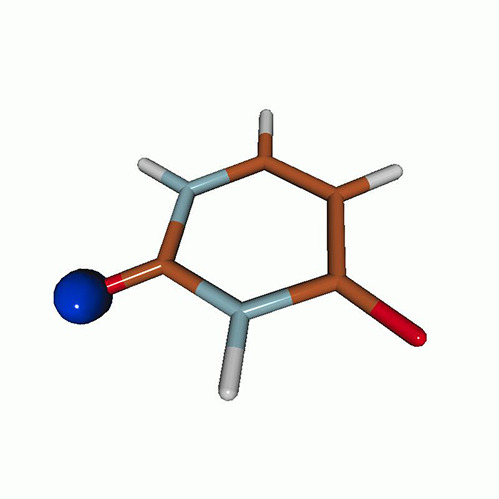	0.67	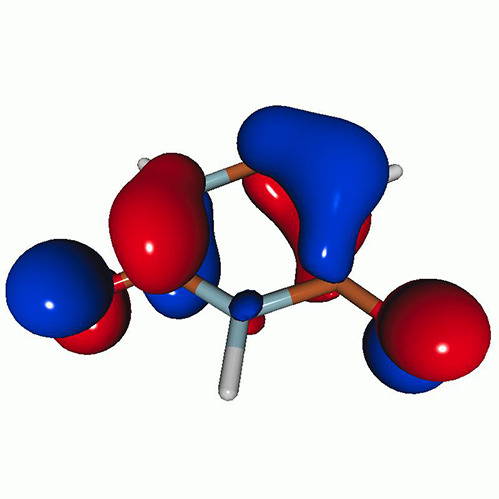
532.96	0.018 6	*β*	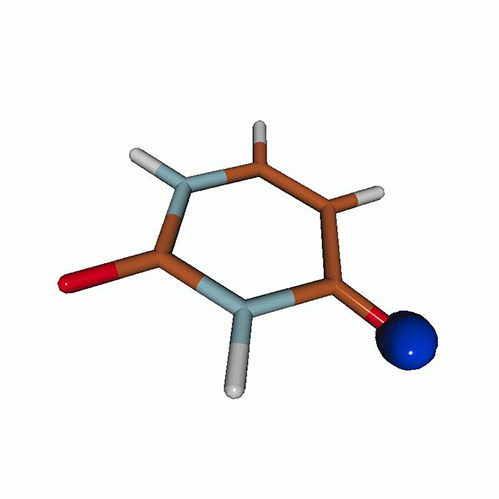	0.74	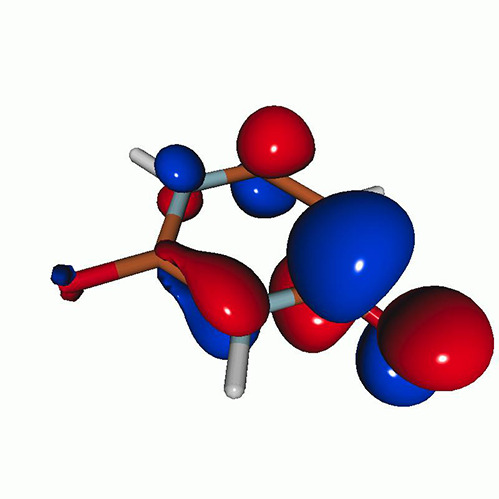
534.74	0.015 5	*β*	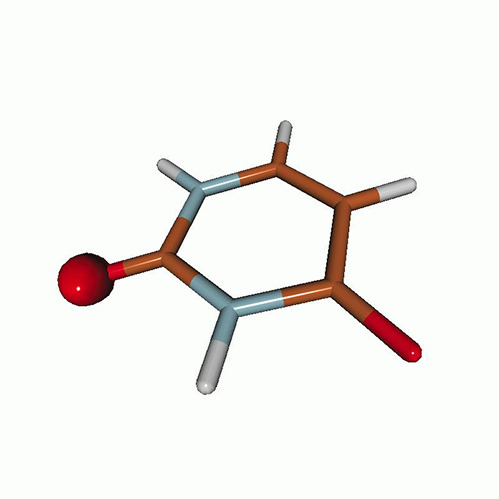	0.80	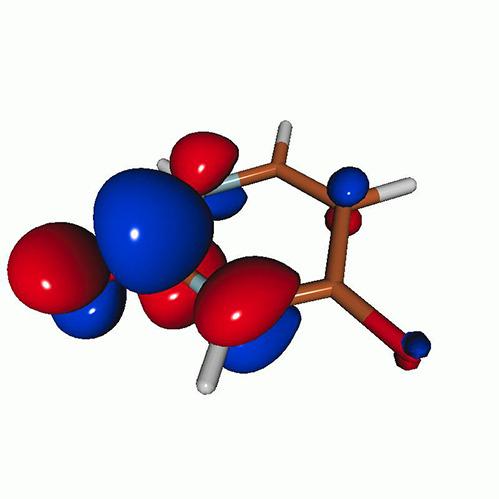
535.70	0.007 6	*α*	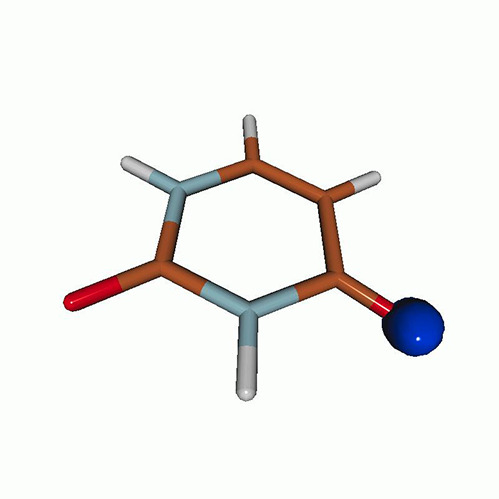	0.77	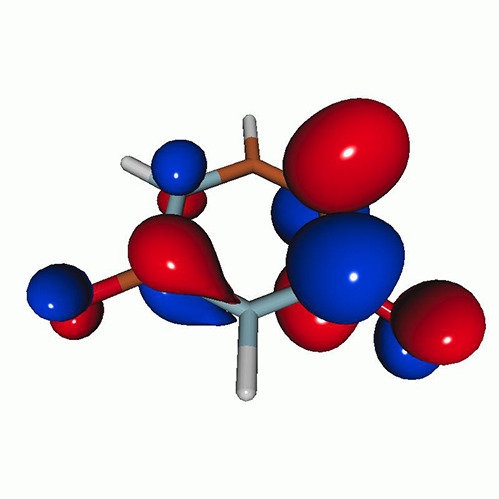
535.88	0.008 5	*α*	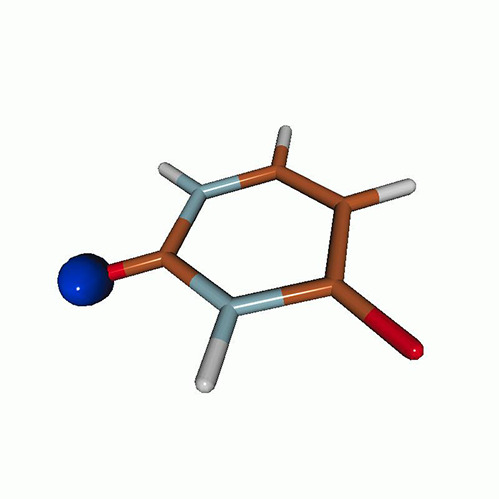	0.76	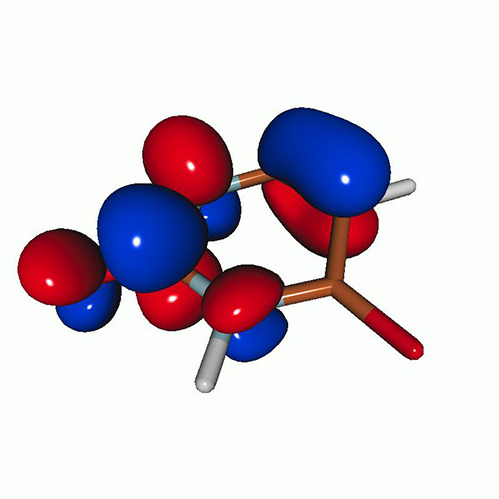

**TABLE X.** Uracil. LSOR-CCSD/6-311++G^**^ NTOs of the O_1*s*_ core excitations from the T_1_($\pi \pi^{*}$) state at the FC geometry (NTO isosurface is 0.05).

$E^{\text{ex}}$ (eV)	Osc. strength	Spin	Hole	$\sigma_{K}^{2}$	Particle
529.81	0.021 2	*β*	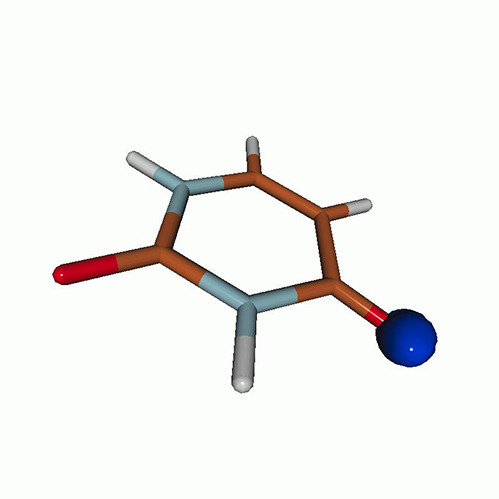	0.79	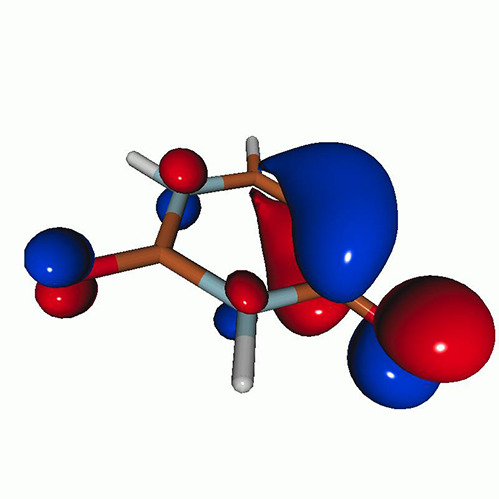
532.39	0.011 5	*β*	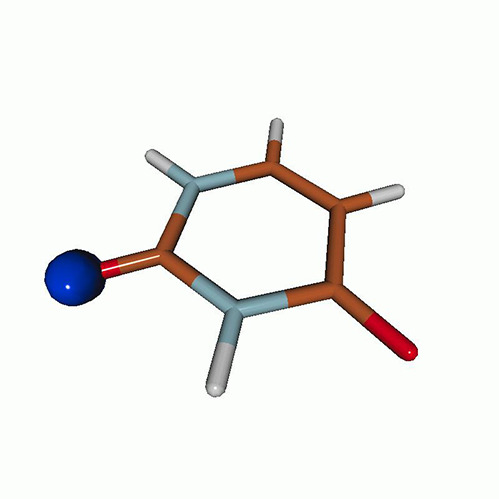	0.78	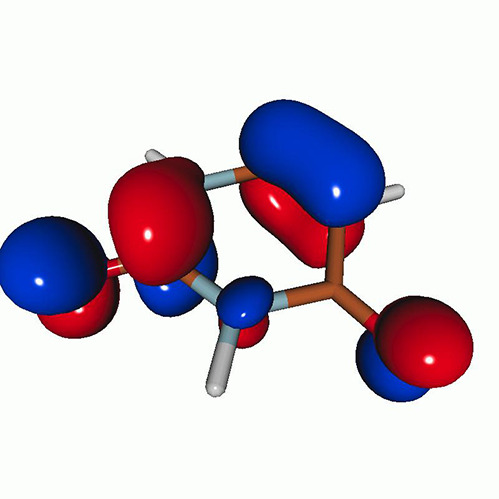
534.15	0.018 7	*α*	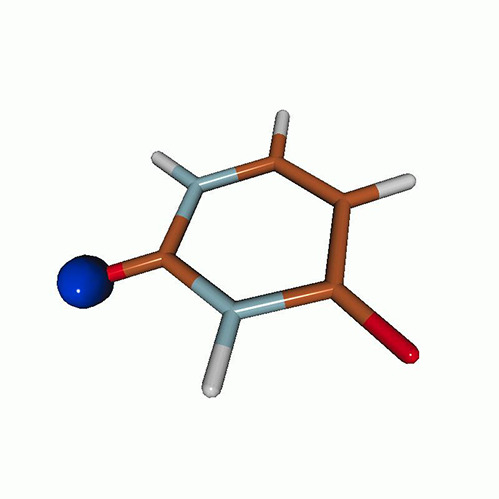	0.76	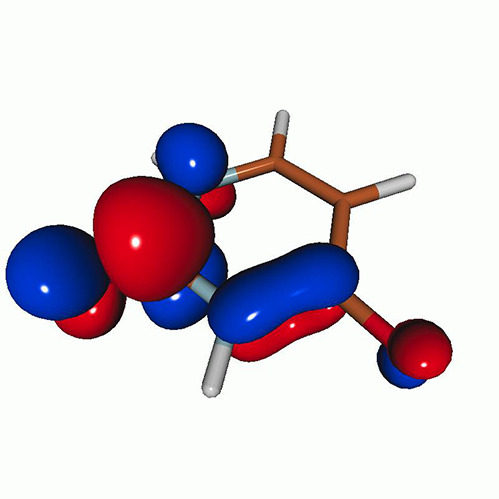
535.09	0.010 0	*α*	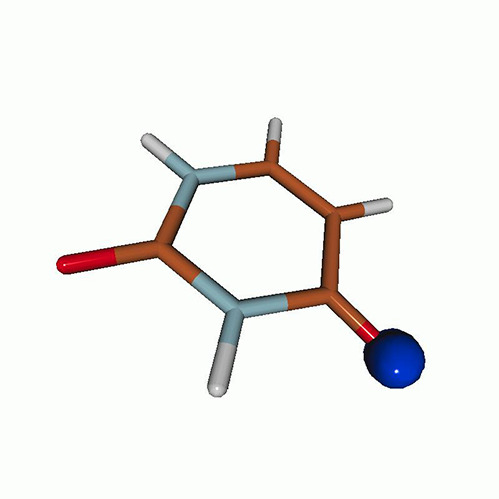	0.73	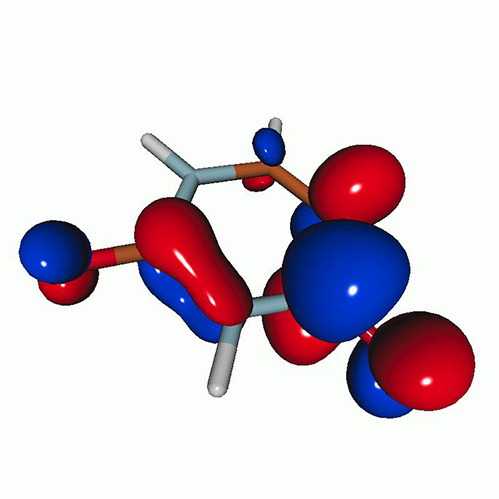
535.58	0.006 2	*β*	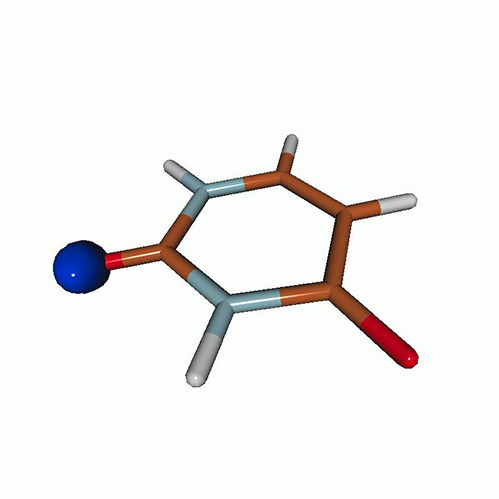	0.77	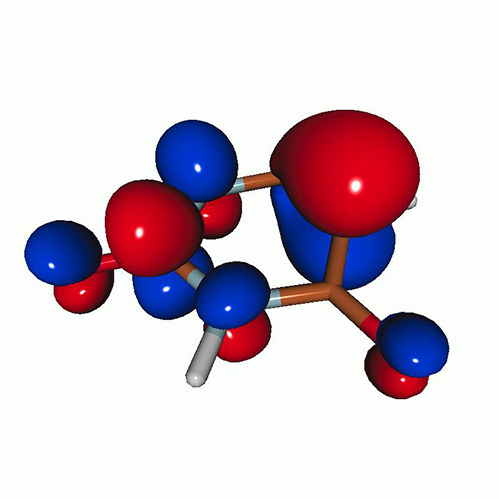
535.61	0.008 1	*β*	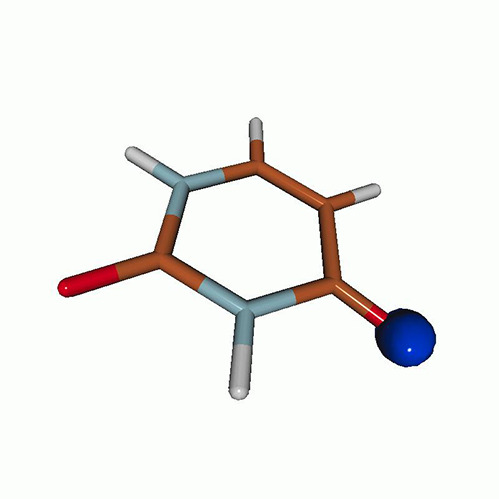	0.72	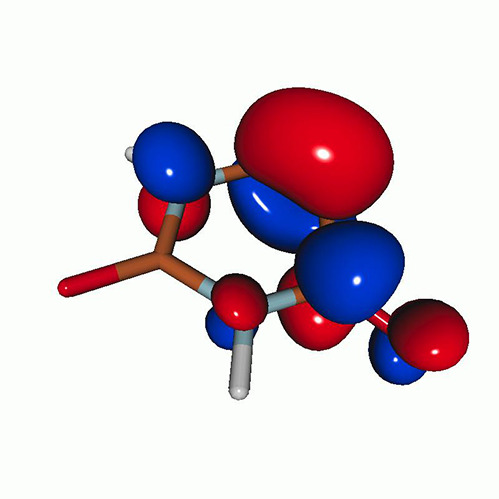

The CVS-EOM-CCSD spectrum of S_1_($n\pi^{*}$) exhibits a relatively intense peak at 528.02 eV, and tiny peaks at 532.40 and 532.52 eV. The intense peak is due to transition from the 1*s* orbital of O4 to SOMO, which is a lone-pair-type orbital localized on O4. The tiny peak at 532.40 eV is assigned to the transition to SOMO from the 1*s* orbital of O2, whereas the peak at 532.52 eV is assigned to a transition with multiply excited character. The LSOR-CCSD spectrum exhibits the strong core-to-SOMO transition peak at 526.39 eV, which is red-shifted from the corresponding CVS-EOM-CCSD one by 1.63 eV. As Table VIII shows that the peak at 534.26 eV is due to transition from the 1*s* orbital of O2 to a $\pi^{*}$ orbital, and it corresponds to the second peak in the ground-state spectrum. In the S_1_($n\pi^{*}$) XAS spectrum, there is no peak corresponding to the first band in the ground-state spectrum, there assigned to the O4 1 $s\to \pi^{*}$ transition. This suggests that this transition is suppressed by the positive charge localized on O4 in the S_1_($n\pi^{*}$) state.

The S_1_($n\pi^{*}$) state from LSOR-CCSD is spin-contaminated, with $\langle S^{2}\rangle =1.033$. The spectra of S_1_($n\pi^{*}$) yielded by LSOR-CCSD [panel (a)] and by HSOR-CCSD [panel (c)] are almost identical. This is not too surprising, as the spectra of S_1_($n\pi^{*}$) and T_2_($n\pi^{*}$) from CVS-EOM-CCSD are also almost identical. This is probably a consequence of small exchange interactions in the two states (the singlet and the triplet) due to negligible spatial overlap between the lone pair (n) and $\pi^{*}$ orbitals.

In the CVS-EOM-CCSD spectrum of S_2_($\pi \pi^{*}$), see panel (b), the peaks due to the core-to-SOMO (*π*) transitions from O4 and O2 occur at 527.50 and 531.87 eV, respectively. The additional peak at 531.99 eV is assigned to a transition with multiple electronic excitation. In the LSOR-CCSD spectrum, the core-to-SOMO peaks appear at 530.16 and 530.54 eV, respectively.

As shown in Table IX, we assign the peaks at 532.96 and 534.74 eV in the LSOR-CCSD spectrum to transitions from the 1*s* orbitals of the two oxygens to the $\pi^{*}$ orbital, which is half occupied in S_2_($\pi \pi^{*}$). The NTO analysis reveals that they correspond to the first and second peak of the ground-state spectrum. Note that $\left\langle S^{2}\right\rangle$ = 1.326 for the S_2_($\pi \pi^{*}$) state obtained from LSOR-CCSD.

In the HSOR-CCSD spectrum of the S_2_($\pi \pi^{*}$) state [which is equal to the LSOR-CCSD spectrum of the T_1_($\pi \pi^{*}$) state in panel (d)], the peaks of the core-to-SOMO (*π*) transitions from O4 and O2 appear at 529.81 and 532.39 eV, respectively (see Table X). They are followed by transitions to the half-occupied $\pi^{*}$ orbital at 534.15 and 535.09 eV, respectively. In contrast to what we observed in the S_1_($n\pi^{*}$) spectra, the LSOR-CCSD and HSOR-CCSD spectra of the S_2_($\pi \pi^{*}$) state are qualitatively different. This can be explained, again, in terms of importance of the exchange interactions in the initial and final states. On one hand, there is a stabilization of the T_1_($\pi \pi^{*}$) (initial) state over the S_2_($\pi \pi^{*}$) state by exchange interaction as the overlap between the *π* and $\pi^{*}$ orbitals is not negligible. The exchange interaction between the strongly localized core-hole orbital and the half-occupied valence/virtual orbital in the final core-excited state, on the other hand, is expected to be small.

**FIG. 7.**
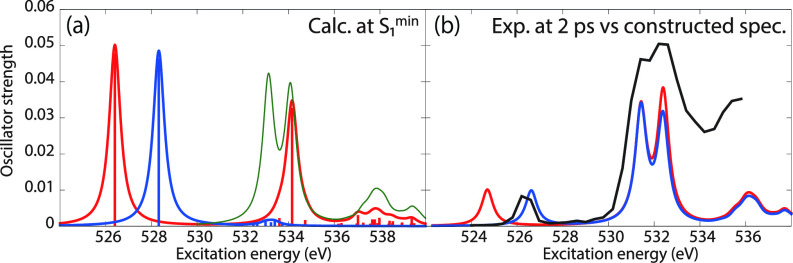
Thymine. (a) Oxygen K-edge NEXAFS in the S_1_(n$\pi^{*}$) state at its potential energy minimum. Blue: CVS-EOM-CCSD. Red: LSOR-CCSD. Thin green line: ground-state spectrum at the FC geometry. (b) Thick black: Experimental spectrum at the delay time of 2 ps.^21^ Blue: computational spectrum made from the blue and green curves of (a), shifted by −1.7 eV. Red: computational spectrum made from the red and green curves of (a), shifted by −1.7 eV. The blue and red curves from (a) were scaled by 0.2 in (b). The ground-state spectrum from (a) was scaled by 0.8 in (b). FWHM of the Lorentzian convolution function is 0.6 eV.

To evaluate the accuracy of the excited-state XAS spectra from CVS-EOM-CCSD and LSOR-CCSD, we also calculated the XAS spectra of the S_1_(n$\pi^{*}$) state of thymine at the potential energy minimum of S_1_(n$\pi^{*}$), see panel (a) of Fig. 7. For construction of the surface cut of the theoretical absorption spectra, we chose FWHM of 0.6 eV for the Lorentzian convolution function. Panel (b) shows the spectra of S_1_(n$\pi^{*}$) multiplied by 0.2 and added to the ground-state spectrum multiplied by 0.8. These factors 0.2 and 0.8 were chosen for the best fit with the experimental spectrum. A surface cut of the experimental TR-NEXAFS spectrum at the delay time of 2 ps (Ref. 21) is also shown in panel (b) of Fig. 7. The reconstructed computational spectra are shifted by −1.7 eV. In the experimental spectrum, the core-to-SOMO transition peak occurs at 526.4 eV. In the reconstructed theoretical spectrum, the core-to-SOMO transition peaks appear at 526.62 and 524.70 eV, for CVS-EOM-CCSD and LSOR-CCSD, respectively. Thus, the CVS-EOM-CCSD superposed spectrum agrees slightly better with experiment than the LSOR-CCSD spectrum. Nonetheless, the accuracy of the LSOR-CCSD spectrum is quite reasonable, as compared with the experimental spectrum.

Due to the lack of experimental data, not much can be said about the accuracy of CVS-EOM-CCSD and LSOR-CCSD/HSOR-CCSD for core excitations from a triplet excited state in uracil and thymine. Furthermore, we are unable to unambiguously clarify, using uracil and thymine as model system, which of the two methods, LSOR-CCSD or HSOR-CCSD, should be considered more reliable when they give qualitatively different spectra for the singlet excited states.

**FIG. 8.**
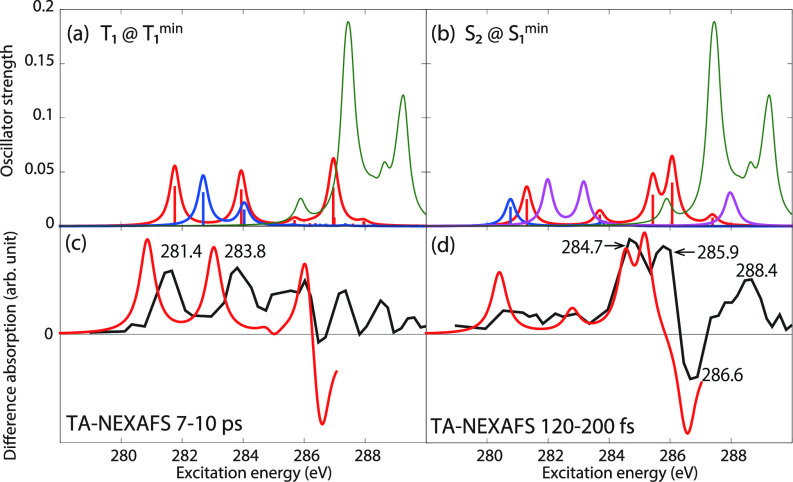
Acetylacetone. Carbon K-edge NEXAFS from the T_1_($\pi \pi^{*}$) (a) and S_2_($\pi \pi^{*}$) (b) states. The spectra of T_1_($\pi \pi^{*}$) were computed at the potential energy minimum of T_1_($\pi \pi^{*}$). The spectra of S_2_($\pi \pi^{*}$) were computed at the potential energy minimum of S_1_(n$\pi^{*}$). Blue: CVS-EOM-CCSD. Red: LSOR-CCSD. Magenta: HSOR-CCSD. Green: Ground-state spectrum at the FC geometry. (c), (d) Black: Experimental transient absorption spectra at the delay times of 7–10 ps and 120–200 fs,^22^ respectively. Red: computational transient absorption spectra made from the red and the green curves of (a) and (b), respectively, shifted by −0.9 eV as the spectrum of the ground state [see panel (b) of Fig. 5]. The red curves of panels (a) and (b) were scaled by 0.75 and from these, the green ground-state spectrum, scaled by 0.25, was subtracted. FWHM of the Lorentzian convolution function is 0.4 eV for panels (a) and (b), 0.6 eV for panels (c) and (d), respectively. Basis set: 6-311++G^**^.

Therefore, we turn our attention to the carbon K-edge spectra of acetylacetone and show, in Fig. 8, the spectra obtained using CVS-EOM-CCSD (blue), LSOR-CCSD (red), and HSOR-CCSD (magenta) for the T_1_($\pi \pi^{*}$) [panel (a)] and S_2_($\pi \pi^{*}$) [panel (b)] states. The T_1_($\pi \pi^{*}$) spectra were obtained at the potential energy minimum of T_1_($\pi \pi^{*}$). The spectra of S_2_($\pi \pi^{*}$) were calculated at the potential energy minimum of the S_1_(n$\pi^{*}$) state. In doing so, we assume that the nuclear wave packet propagates on the S_2_($\pi \pi^{*}$) surface toward the potential energy minimum of the S_1_(n$\pi^{*}$) surface. Note that CVS-EOM-CCSD does not describe all the core excitations from a valence-excited state (see Fig. 2). In panels (c) and (d), the LSOR-CCSD spectra were multiplied by 0.75 and subtracted from the ground-state spectrum, scaled by 0.25, and superposed to the surface cuts of the experimental transient-absorption NEXAFS at delay times 7–10 ps and 120–200 fs, respectively. The calculated transient-absorption spectra were shifted by −0.9 eV, i.e., by the same amount as the spectrum of the ground state [see panel (b) of Fig. 5]. For construction of the surface cut of the theoretical transient-absorption spectra, we used FWHM of 0.6 eV for the Lorentzian convolution function. The scaling factors values 0.75 and 0.25 were chosen to yield the best fit with the experimental spectra. The NTOs of the core excitations from T_1_($\pi \pi^{*}$) and S_2_($\pi \pi^{*}$) are shown in Tables XI and XII, respectively. In the experimental study,^22^ it was concluded that S_2_($\pi \pi^{*}$) is populated at the shorter timescale, whereas at the longer timescale it is T_1_($\pi \pi^{*}$) that becomes populated.

**TABLE XI.** Acetylacetone. LSOR-CCSD/6-311++G^**^ NTOs of the C_1*s*_ core excitations from the T_1_ state at the potential energy minimum (NTO isosurface is 0.05).

$E^{\text{ex}}$ (eV)	Osc. strength	Spin	Hole	$\sigma_{K}^{2}$	Particle
281.76	0.034 7	*β*	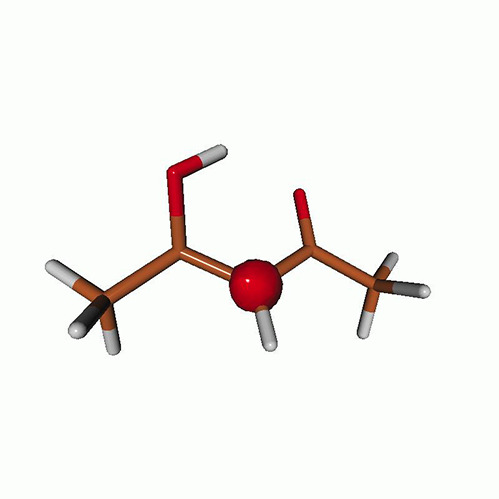	0.86	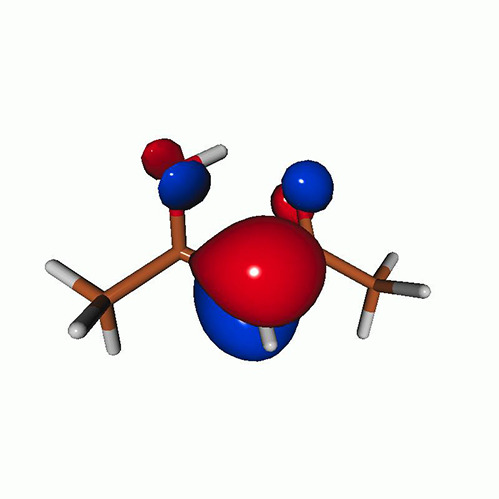
283.94	0.031 8	*β*	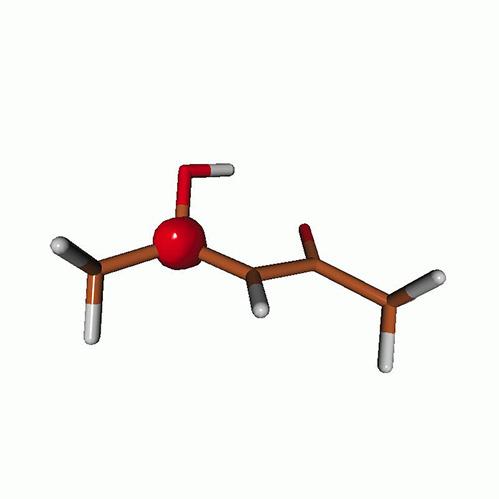	0.84	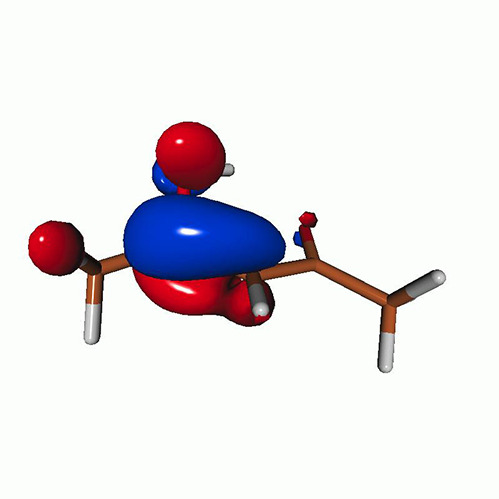
285.69	0.003 6	*β*	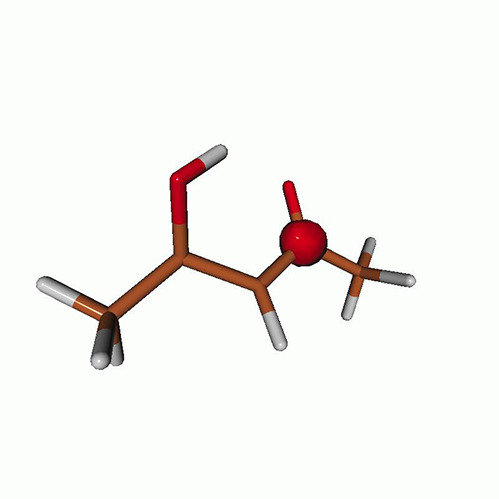	0.72	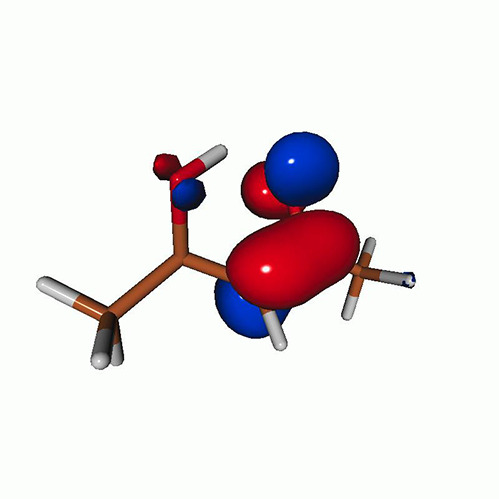
286.96	0.033 4	*α*	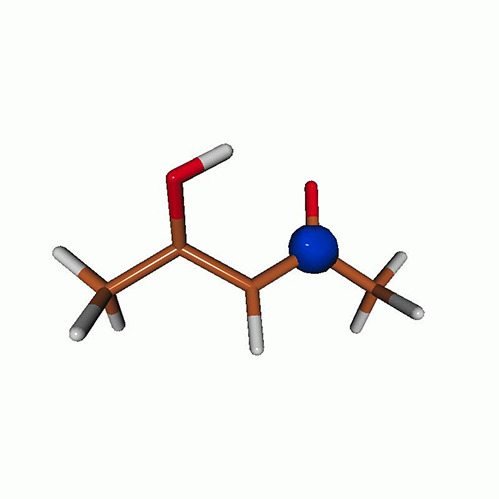	0.65	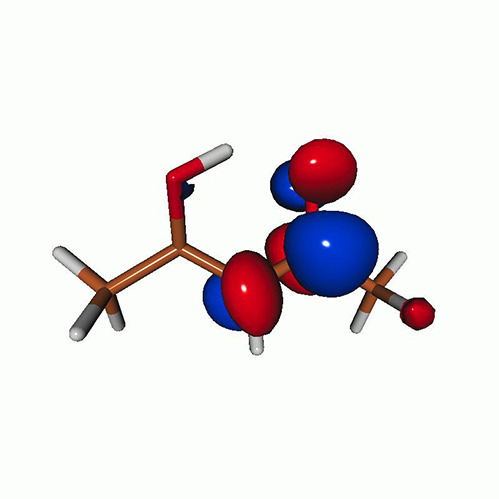
		*β*	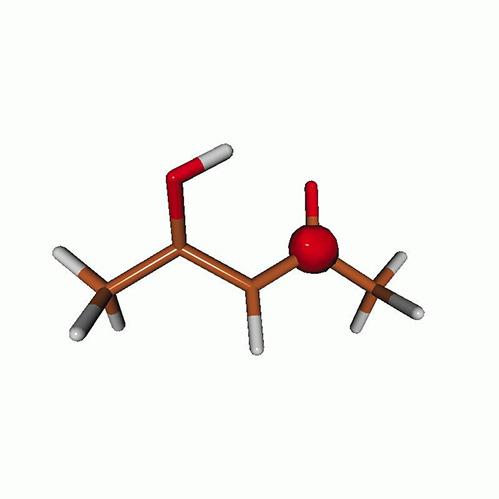	0.14	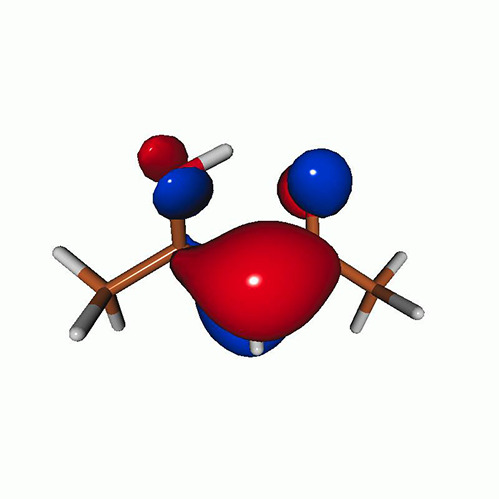

**TABLE XII.** Acetylacetone. LSOR-CCSD/6-311++G^**^ NTOs of the C_1*s*_ core excitations from the S_2_ state at the potential energy minimum of S_1_ (NTO isosurface is 0.05).

$E^{\text{ex}}$ (eV)	Osc. strength	Spin	Hole	$\sigma_{K}^{2}$	Particle
281.30	0.022 8	α	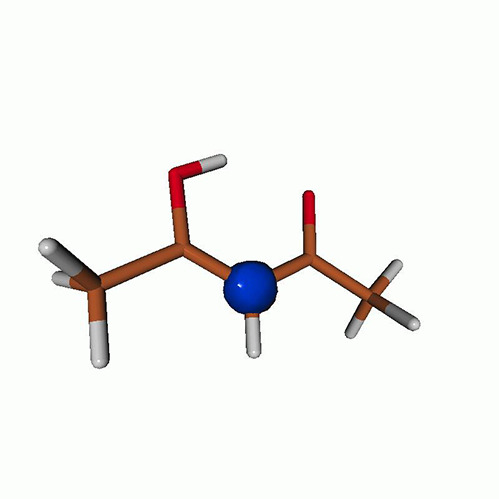	0.77	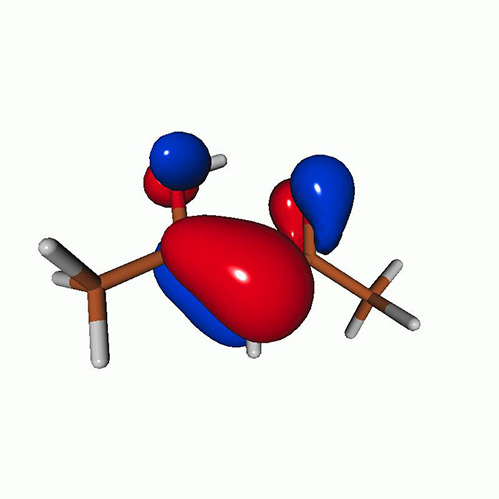
283.69	0.008 5	α	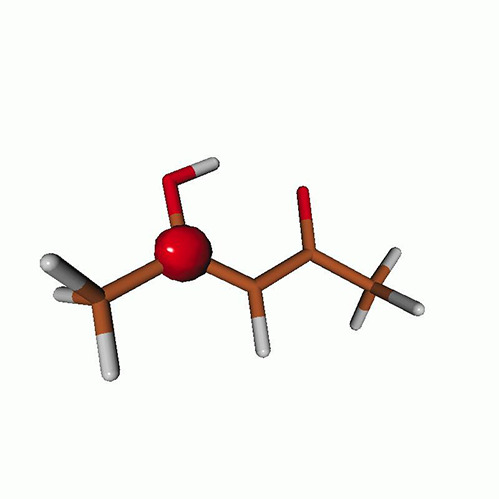	0.71	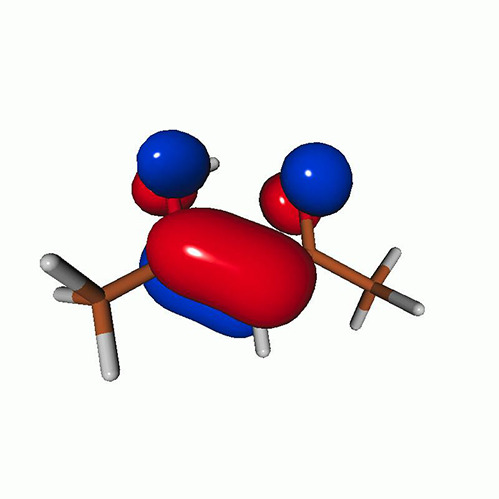
285.43	0.026 9	β	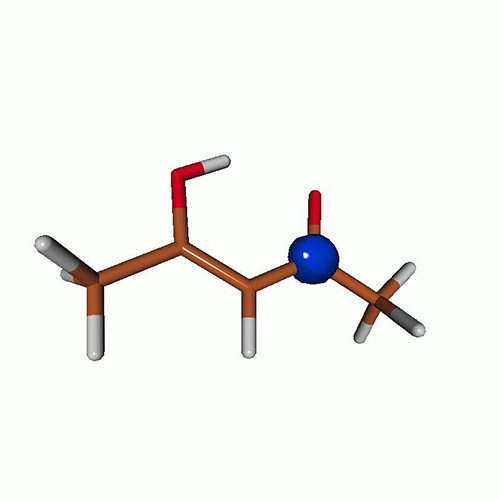	0.76	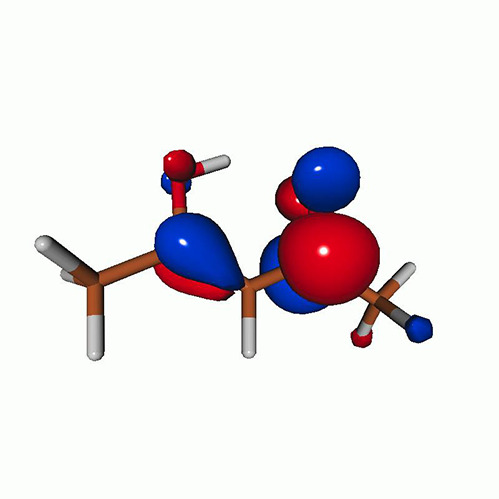
286.07	0.038 1	β	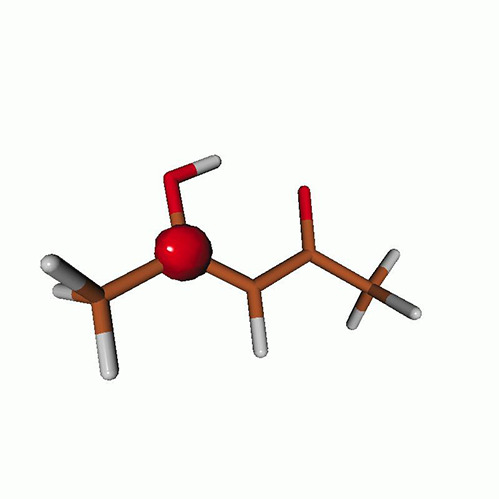	0.76	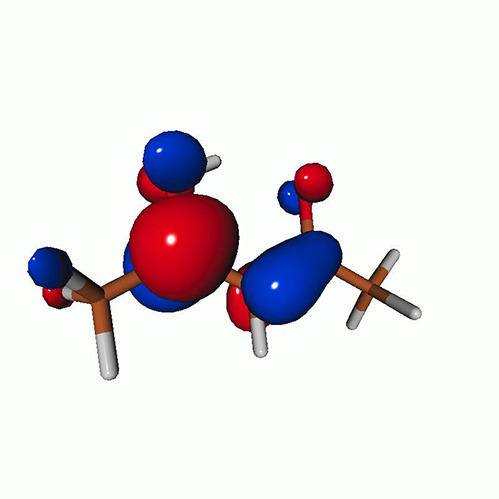
287.39	0.005 7	β	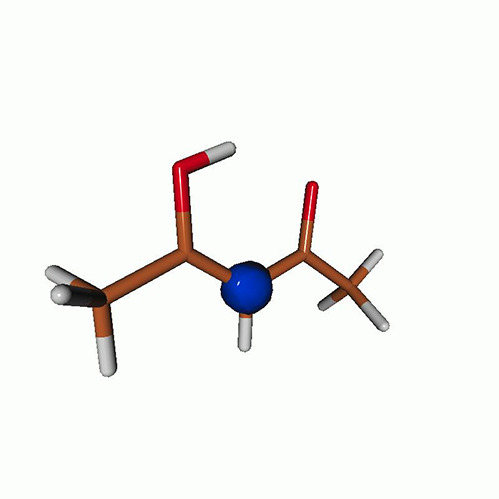	0.64	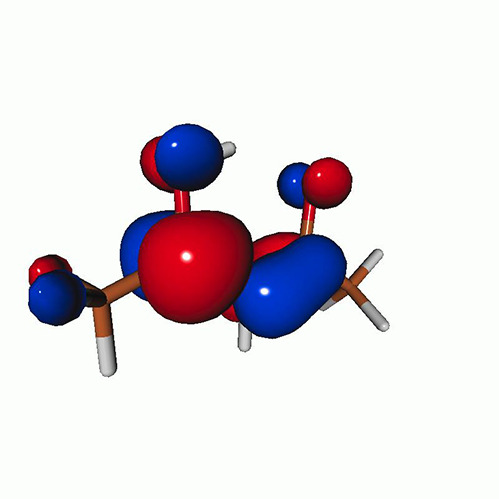

The surface cut of the experimental transient-absorption spectra at longer times (7–10 ps) features two peaks at 281.4 and 283.8 eV. In panel (a) of Fig. 8, the CVS-EOM-CCSD spectrum of T_1_($\pi \pi^{*}$) shows the core-to-SOMO transition peaks at 282.69 and 284.04 eV, whereas the LSOR-CCSD ones appear at 281.76 and 283.94 eV. The LSOR-CCSD spectrum also shows a peak corresponding to a transition from C4 to the half-occupied $\pi^{*}$ orbital at 286.96 eV (see Table XI). The separation of 2.4 eV between the two core-to-SOMO peaks in the experiment is well reproduced by LSOR-CCSD. Spin contamination is small, $\left\langle S^{2}\right\rangle$ = 2.004 for the T_1_($\pi \pi^{*}$) state obtained using LSOR-CCSD. Therefore, it is safe to say, that LSOR-CCSD accurately describes core excitations from the low-lying triplet states.

The surface cut of the transient-absorption spectra at shorter times, 120–240 fs, features relatively strong peaks at 284.7, 285.9 and a ground-state bleach at 286.6 eV. The CVS-EOM-CCSD spectrum of the S_2_($\pi \pi^{*}$) state shows the core-to-SOMO peak at 280.77. The LSOR-CCSD spectrum (red) has core-to-SOMO transition peaks at 281.30 and 283.69 eV, plus the peaks due to the transitions from the core of C2, C4, and C3 to the half-occupied $\pi^{*}$ orbital at 285.43, 286.07 and 287.39 eV, respectively (see Table XII). Note that the peaks at 285.43 and 286.07 eV correspond to the main degenerate peaks of the ground-state spectrum, as revealed by inspection of the NTOs. The HSOR-CCSD spectrum (magenta) exhibits the core-to-SOMO transition peaks at 281.99 and 283.17 eV, followed by only one of the quasi-degenerate peaks corresponding to transitions to the half-occupied $\pi^{*}$ orbital, at 287.95 eV. Since the experimental surface-cut spectrum does not clearly show the core-to-SOMO transition peaks, it is difficult to assess the accuracy of these peaks as obtained in the calculations. When it comes to the experimental peaks at 284.7 and 285.9 eV, only LSOR-CCSD reproduces them with reasonable accuracy. The experimental peak at 288.4 eV is not reproduced. In the case of acetylacetone, the HSOR-CCSD approximation fails to correctly mimic the spectrum of S_2_($\pi \pi^{*}$), since it does not give the peaks at 284.7 and 285.9 eV. The differences between LSOR-CCSD and HSOR-CCSD spectra for S_2_($\pi \pi^{*}$) can be rationalized as done for uracil.

We emphasize that the assignment of the transient absorption signal at shorter time to S_2_($\pi \pi^{*}$) is based on peaks assigned to transitions to the $\pi^{*}$ orbitals (almost degenerate in the ground state), which cannot be described by CVS-EOM-CCSD (see Fig. 2 in Sec. II A).

On the basis of the above analysis, we conclude that, despite spin contamination, LSOR-CCSD describes the XAS of singlet valence-excited states with reasonable accuracy. LSOR-CCSD could even be used as benchmark for other levels of theory, especially when experimental TR-XAS spectra are not available.

We conclude this section by analyzing the MOM-TDDFT results for the transient absorption. As seen in Secs. III A and III B, ADC(2) and TDDFT/B3LYP yield reasonable results for the lowest-lying core-excited states and for the valence-excited states of interest in the nuclear dynamics. The next question is thus whether MOM-TDDFT/B3LYP can reproduce the main peaks of the time-resolved spectra with reasonable accuracy. We attempt to answer this question by comparing the MOM-TDDFT/B3LYP spectra of thymine and acetylacetone with the surface cuts of the experimental spectra.

**FIG. 9.**
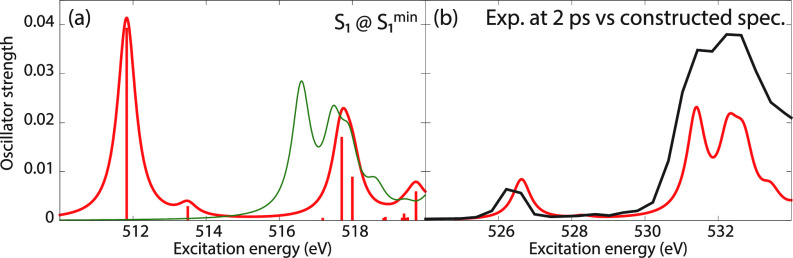
(a) Red: Oxygen K-edge NEXAFS for thymine in the S_1_(n$\pi^{*}$) state calculated at the MOM-TDDFT/B3LYP/6-311++G^**^ level at the potential energy minimum. Green: Ground-state spectrum. (b) Black: Experimental spectrum at the delay time of 2 ps,^21^ Red: computational spectrum made from the red and the green curves of (a), shifted by +14.8 eV. The red curve of (a) was scaled by 0.2. The green curve of (a) was scaled by 0.8. FWHM of the Lorentzian convolution function is 0.6 eV.

The MOM-TDDFT/B3LYP O K-edge NEXAFS spectrum of thymine in the S_1_(n$\pi^{*}$) state is shown in Fig. 9, panel (a). For construction of the surface cut of the theoretical absorption spectra, we used FWHM of 0.6 eV for the Lorentzian convolution function. A theoretical surface cut spectrum was constructed as sum of the MOM-TDDFT spectrum and the standard TDDFT spectrum of the ground state, scaled by 0.2 and 0.8, respectively. This is shown in panel (b), together with the experimental surface cut spectrum at 2 ps delay.^21^ The MOM-TDDFT/B3LYP peaks due to the core transitions from O4 and O2 to SOMO (n) are found at 511.82 and 513.50 eV, respectively. The peak corresponding to the first main peak of the ground-state spectrum is missing, and the one corresponding to the second main peak in the ground state appears at 517.71 eV. These features are equivalent to what we observed in the LSOR-CCSD case (see Fig. 7). Thus, the separation between the core-to-SOMO peak and the ground-state main peaks is accurately reproduced.

**FIG. 10.**
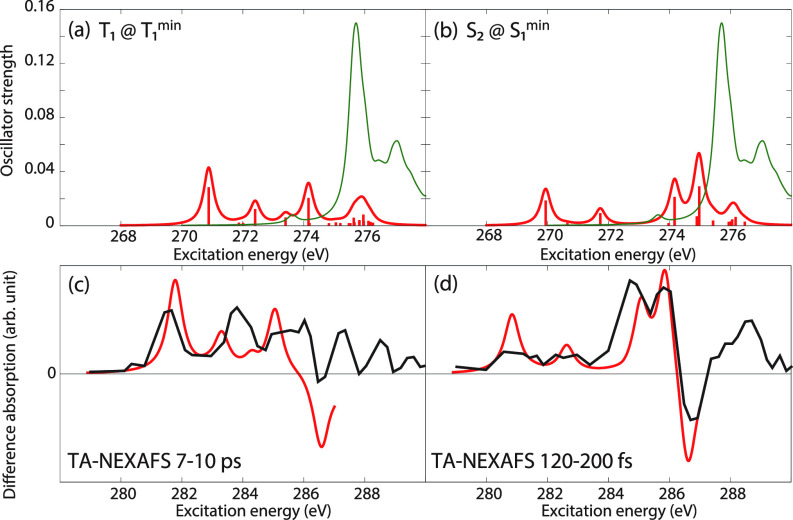
(a) and (b) Carbon K-edge NEXAFS for acetylacetone in the T_1_($\pi \pi^{*}$) and S_2_($\pi \pi^{*}$) states calculated at the MOM-TDDFT/B3LYP/6-311++G^**^ level at the potential energy minima of T_1_($\pi \pi^{*}$) and S_1_(n$\pi^{*}$), respectively. The green curve is the ground-state spectrum. In panels (c) and (d), the experimental transient absorption spectra at delay times of 7–10 ps and 120–200 fs are reported with black lines.^22^ In red are the computational transient absorption spectra reconstructed from the red and green curves of panels (a) and (b), respectively, shifted by +10.9 eV. The red curves of (a) and (b) were scaled by 0.75, and subtracted from the green curves, which were scaled by 0.25. FWHM of the Lorentzian convolution function is 0.4 eV for panels (a) and (b), 0.6 eV for panels (c) and (d), respectively.

Next, we consider the carbon K-edge spectra of acetylacetone in the T_1_($\pi \pi^{*}$) [at the minimum of T_1_($\pi \pi^{*}$)] and S_2_($\pi \pi^{*}$) [at the minimum of S_1_(n$\pi^{*}$)] states, as obtained from MOM-TDDFT. They are plotted in panels (a) and (b) of Fig. 10, respectively. Surface cuts of the transient-absorption NEXAFS spectra were constructed by subtracting the TDDFT spectrum, scaled by 0.25, with the MOM-TDDFT spectra scaled by 0.75. For this construction, we convoluted the oscillator strengths with a Lorentzian function (FWHM = 0.6 eV) and chose the factors 0.75 and 0.25 for the best fit with the experimental spectra. They are superposed with those from experiment at delay times of 7–10 ps and 120–200 fs in Fig. 10, panels (c) and (d). The MOM-TDDFT spectrum of T_1_($\pi \pi^{*}$) exhibits the core-to-SOMO transition peaks at 270.88 and 272.41 eV. A peak due to the transition to the half-occupied $\pi^{*}$ orbital occurs at 274.16 eV. All peaks observed in the LSOR-CCSD spectrum were also obtained by MOM-TDDFT. The fine structure of the surface-cut transient absorption spectrum is qualitatively reproduced.

The MOM-TDDFT spectrum of S_2_($\pi \pi^{*}$) exhibits the core-to-SOMO($\pi^{*}$) transition peaks at 269.94 and 271.73 eV. The peaks due to the transitions to the half-occupied $\pi^{*}$ orbital appear at 274.17 and 274.98 eV. The reconstructed transient-absorption spectrum agrees well with the experimental surface-cut spectrum.

## IV. SUMMARY AND CONCLUSIONS

We have analyzed the performance of different single-reference electronic structure methods for excited-state XAS calculations. The analysis was carried out in three steps. First, we compared the results for the ground-state XAS spectra of uracil, thymine, and acetylacetone computed using CVS-ADC(2), CVS-EOM-CCSD, and TDDFT/B3LYP, and with the experimental spectra. Second, we computed the excitation energies of the valence-excited states presumably involved in the dynamics at ADC(2), EOM-EE-CCSD, and TDDFT/B3LYP levels, and compared them with the experimental data from EELS and UV absorption. Third, we analyzed different protocols for the XAS spectra of the lowest-lying valence-excited states based on the CCSD ansatz, namely, regular CVS-EOM-CCSD for transitions between excited states, and EOM-CCSD applied on the excited-state reference state optimized imposing the MOM constraint. The results for thymine and acetylacetone were evaluated by comparison with the experimental time-resolved spectra. Finally, the performance of MOM-TDDFT/B3LYP for TR-XAS was evaluated, again on thymine and acetylacetone, by comparison with the LSOR-CCSD and the experimental spectra.

In the first step, we found that CVS-EOM-CCSD reproduces well the entire pre-edge region of the ground-state XAS spectra. On the other hand, CVS-ADC(2) and TDDFT/B3LYP only describe the lowest-lying core excitations with reasonable accuracy, while the Rydberg region is not captured. In the second step, we observed that EOM-EE-CCSD, ADC(2), and TDDFT/B3LYP treat the valence-excited states with a comparable accuracy.

Among the methods analyzed in the third step, only LSOR-CCSD and MOM-TDDFT can reproduce the entire pre-bleaching region of the excited-state XAS spectra for thymine and acetylacetone, despite spin contamination of the singlet excited states. LSOR-CCSD could be used as the reference when evaluating the performance of other electronic structure methods for excited-state XAS, especially if no experimental spectra are available. For the spectra of the spin-singlet states, CVS-EOM-CCSD yields slightly better core → SOMO positions.

We note that the same procedure can be used to assess the performance of other xc-functional or post-HF methods for TR-XAS calculations. We also note that description of an initial state with the MOM algorithm is reasonably accurate only when the initial state has a single configurational wave-function character. The low computational scaling and reasonable accuracy of MOM-TDDFT makes it rather attractive for the on-the-fly calculation of TR-XAS spectra in the excited-state nuclear dynamics simulations.

## SUPPLEMENTARY MATERIAL

See the supplementary material for the NTOs of all core and valence excitations.

## Data Availability

The data that support the findings of this study are available within the article and its supplementary material.
